# Supplementary material for: Twisted superconducting quantum diodes for high fidelity anharmonic qubits
Source: Nat Commun. 2026 Oct 10;17:11503. doi: 10.1038/s41467-026-78376-1 (PMC13745581; doi:10.1038/s41467-026-78376-1)
Supplement: Supplementary file 1 [file 41467_2026_78376_MOESM1_ESM.pdf]

Supplementary Information for: Twisted superconducting quantum diodes for high fidelity anharmonic qubits

Han Zhong[1], Denis Kochan[2,3], Igor Žutić[4], and Yingying Wu[1,*]

[1]Electrical and Computer Engineering, University of Florida, Gainesville, FL 32611, USA

[2]Department of Physics and Center for Quantum Frontiers of Research and Technology (QFort), National Cheng Kung University, Tainan 70101, Taiwan

[3]Institute of Physics, Slovak Academy of Sciences, 84511 Bratislava, Slovakia

[4]Department of Physics, State University of New York at Buffalo, Buffalo, New York 14260, USA

[*]Corresponding author: yingyingwu@ufl.edu

Supplementary Note 1: Device fabrication

First, 5/30 nm Ti/Au bottom electrodes on SiO_2/Si substrate were created through mask laser exposure and photolithography. Then $NbSe_2$ flake from high-quality bulk materials were exfoliated onto the 300 nm SiO_2/Si substrates. For the critical twisting step, a PDMS/PPC transfer method was employed to pick up half of an $NbSe_2$ flake, rotate it by 1° using the transfer stage rotation knob, and stack it onto the remaining half flake. This employs the "tear and stack" methods described in twisted bilayer graphene case [1, 2]. Following this alignment, a second pick-up was performed to collect the complete twisted $NbSe_2$ stack. This pick-up process involved heating the sample stage to $50\,^\circ C$ while bringing PDMS/PPC into contact with $NbSe_2$, then cooling during PDMS/PPC detachment. After the separation, the $NbSe_2$ was picked up by PDMS/PPC. Another layer of $NbSe_2$ was then picked up by PDMS/PPC/$NbSe_2$ by precisely controlling the twisting angle.

The resulted PDMS/PPC/twisted $NbSe_2$ was then transferred onto the prepared bottom metal electrodes with proper alignment. After removing the PDMS/PPC by acetone, the twisted $NbSe_2$ are in good contact with the bottom electrodes. Finally, thin layers of h-BN were transferred onto this structure to protect it from effects of the oxygen environment. All the procedures were carried out inside a glove box, with H_2O of 0.05 ppm (parts per million) and O_2 less than 0.5 ppm.

Supplementary Note 2: Current-voltage characteristics

The current-voltage ($I - V$), characteristics of a twisted $NbSe_2$ were measured under various magnetic field conditions to analyze the field dependence of the critical current. Figure S1 presents data from four values of an in-plane magnetic field, B. Critical current values were determined by fitting the steepest portion of each transition curve and identifying the intersection with the zero-voltage axis. Measurement uncertainties were quantified through bootstrap statistical analysis, wherein each critical current point underwent 50 random resamplings of neighboring data points. This process recalculated voltage gradients for each resampled dataset, identified critical points from maximum

gradient values, and determined standard deviation across these samples. The resulting error margins are displayed on the $I - V$ plots, providing a clear representation of the measurement precision. The uncertainty is typically one order of magnitude below the critical current, demonstrating the reliability of the calculation algorithm when applied to the experimental data. The asymmetry between forward and reverse critical currents I_C^+ and I_C^- observed at different $\mathbf{B}$ strengths indicates the presence of field $\mathbf{B}$-dependent diode efficiency, $\eta(\mathbf{B})$, see Eq. (1) from the main text. This supplementary data provides additional experimental evidence supporting the $\mathbf{B}$- modulation of the supercurrent and η described in main text.

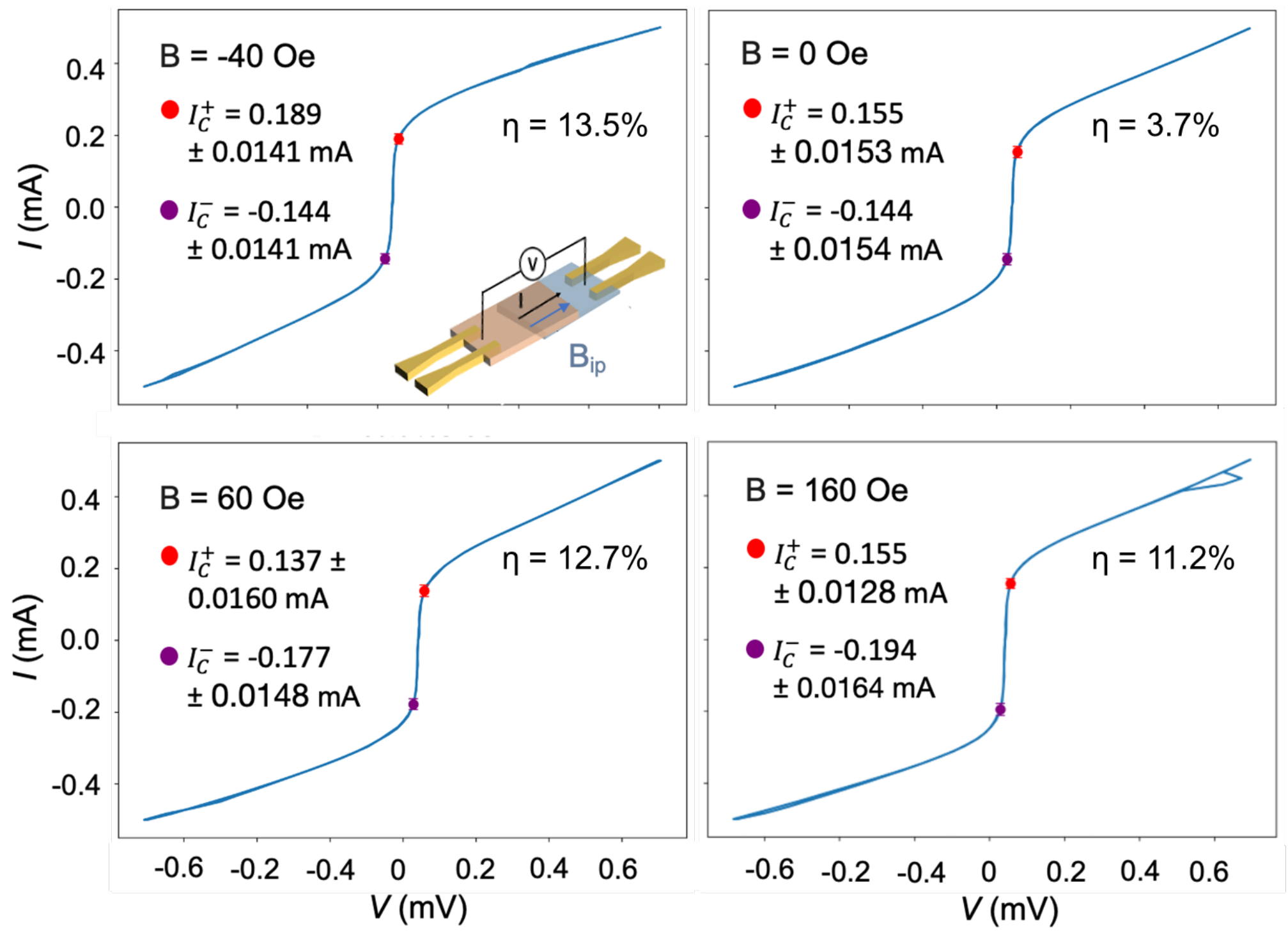

Figure S1: The evolution of the critical currents and diode efficiency with an in-plane magnetic field. The plots show $I - V$ characteristic at $\mathbf{B}$ = -40 Oe, 0 Oe, 60 Oe, and 160 Oe, with the forward (reverse) critical current values I_C^+ (I_C^-) measured and calculated at each field strength, given with the corresponding diode efficiency, η. Inset: Schematic of the experimental setup showing the device geometry and an in-plane magnetic field orientation.

Supplementary Note 3: Critical currents for different devices

Figure S2 reveals how critical currents vary with $\mathbf{B}$ in $NbSe_2$ devices of different thicknesses. An overall 60 nm twisted $NbSe_2$ sample, shown in Figs. S2a and 2b, demon-

strates a small difference in the forward I_C^+ and reverse (I_C^-) critical currents as a function of an in-plane and out-of-plane $\mathbf{B}$, respectively. While this $\mathbf{B}$-anisotropy in the critical currents is small, a change of the sample thickness plays an important role. Conversely, an overall thickness of 30 nm twisted $NbSe_2$ sample depicted in Figs. S2c and S2d exhibits a more pronounced $\mathbf{B}$-anisotropy and a larger and irregular differences between I_C^+ and I_C^-. This thickness-dependent dependence of the nonreciprocal supercurrent response shows a complex behavior of twisted $NbSe_2$ diodes with $\mathbf{B}$.

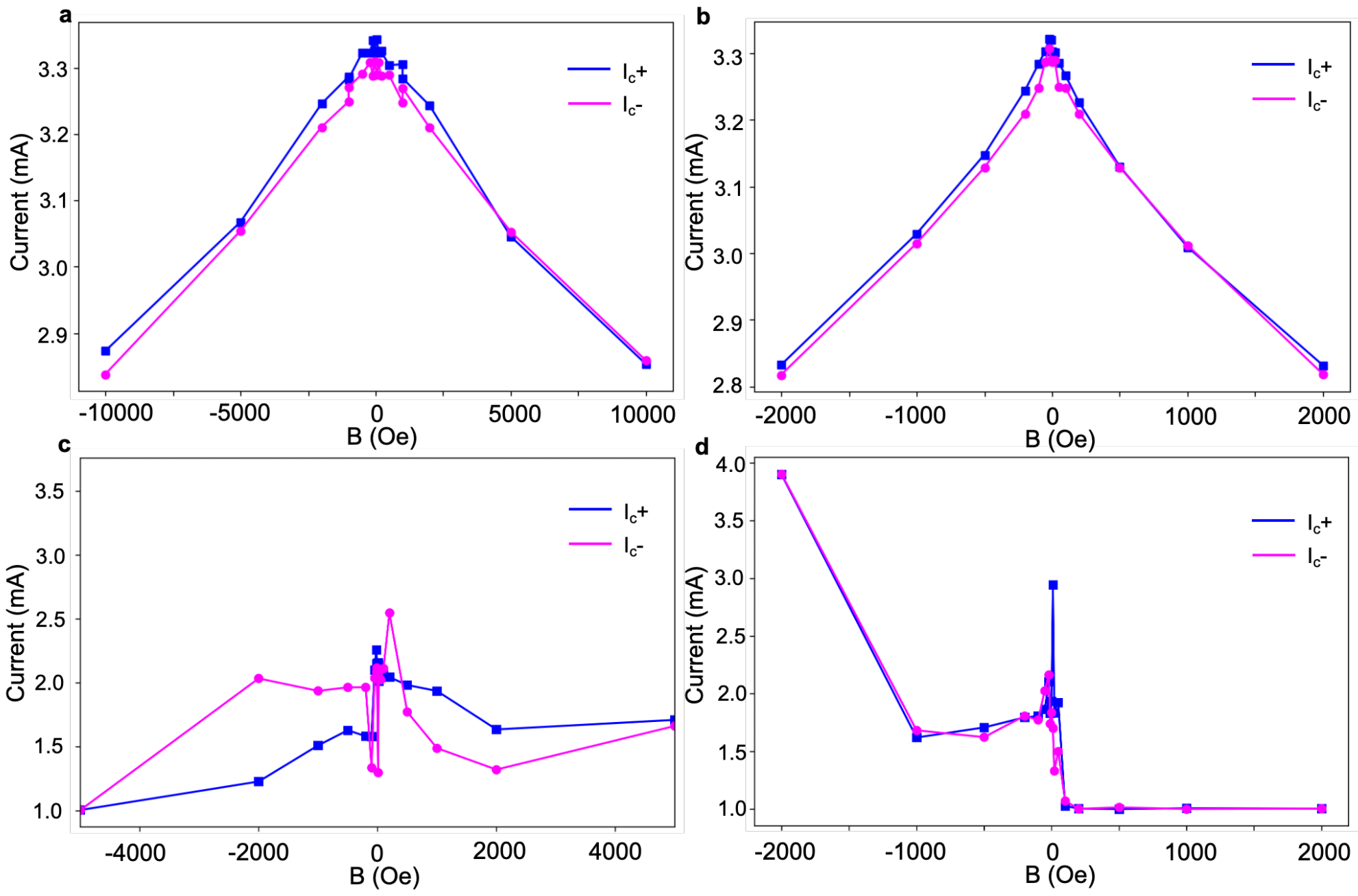

Figure S2: Thickness-dependence of the asymmetry and anisotropy of the critical currents with B. (a) Forward and reverse critical current with in-plane $\mathbf{B}$ and (b) out-of-plane $\mathbf{B}$ for an overall thickness of 60 nm twisted $NbSe_2$. (c) and (d) The same results for an overall thickness of 30 nm twisted $NbSe_2$.

Following the notation from the main text, we model the diode element by an effective current-phase relation (CPR) $I(\varphi) = I_C f(\varphi)$ and define the associated washboard potential $U(\varphi) = E_J F(\varphi)$, such that $f(\varphi) = \partial_\varphi F(\varphi)$ and E_J is the Josephson energy [3]. The parameter η chosen for the energy landscape of the washboard potential $F(\varphi)$ is directly related, one-to-one, to the resulting diode efficiency measured in the device circuit. For our minimal CPR,

$$f(\varphi) = \sin(\varphi - \delta) + \eta, \qquad \delta = \arcsin \eta,$$

the extrema are directly obtained because $\sin(\varphi - \delta) \in [-1, 1]$:

$$I_C^+ = I_C \max_{\varphi} f(\varphi) = I_J(1+\eta), \qquad I_C^- = I_C \min_{\varphi} f(\varphi) = I_J(-1+\eta).$$

Hence $|I_C^-| = I_C(1-\eta)$ for $0 \leq \eta < 1$, and the diode efficiency becomes

$$\eta = \frac{|I_C^+| - |I_C^-|}{|I_C^+| + |I_C^-|} = \frac{(1+\eta) - (1-\eta)}{(1+\eta) + (1-\eta)} = \eta.$$

Therefore, the model parameter in $F(\varphi)$ equals the experimentally defined diode efficiency.

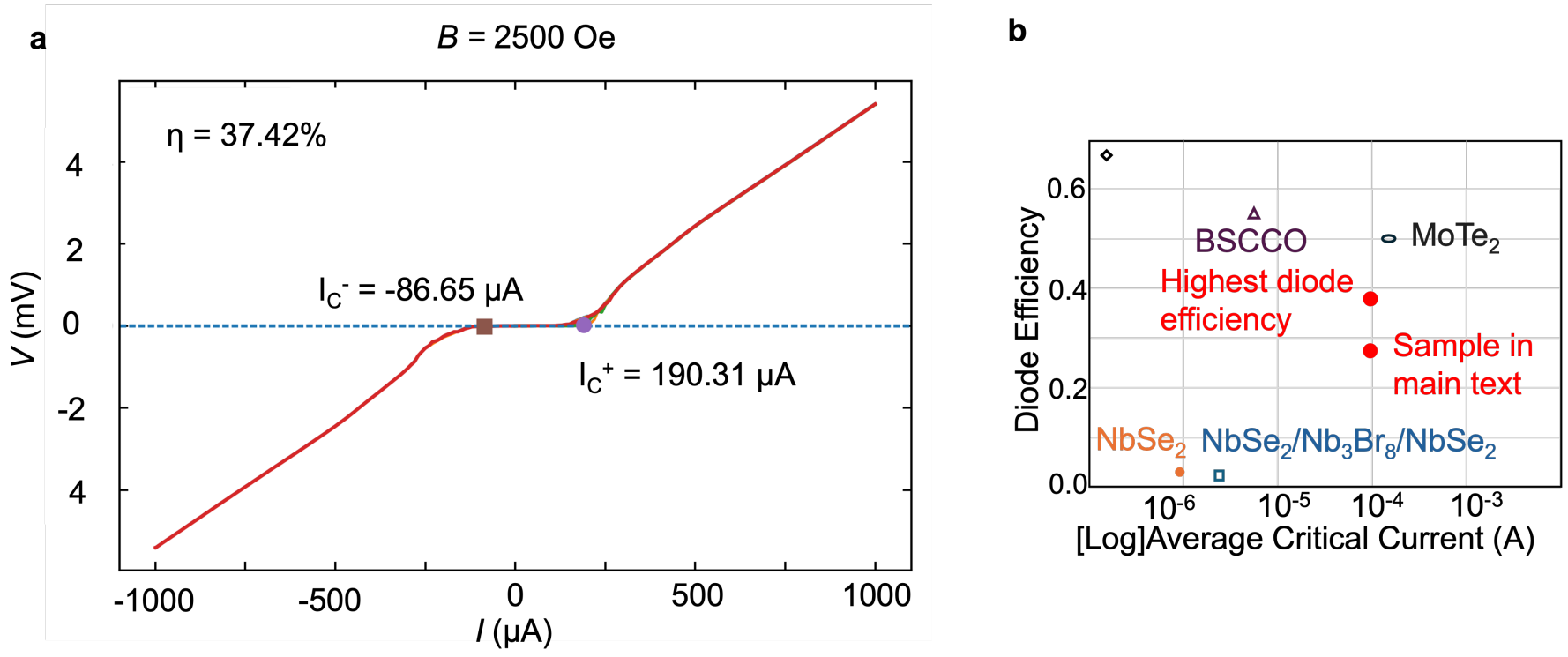

Figure S3: Highest diode efficiency among all measured samples and comparison with previously reported materials. (a) Highest diode efficiency observed at $\mathbf{B}_{\text{oop}} = 2500$ Oe. (b) Comparison with previously reported materials.

Figure S3a shows that the superconducting diode efficiency reaches $\eta = 37.42\%$ at out-of-plane magnetic field $\mathbf{B}_{\text{oop}} = 2500$ Oe, with the critical currents extracted as $I_C^+ = 190.31\ \mu$A and $I_C^- = -86.65\ \mu$A. Figure S3b compares this value with previously reported superconducting diode efficiencies in other systems. Our twisted $NbSe_2$ device exhibits a notably enhanced diode efficiency and is highlighted as the sample discussed in the main text.

We fabricated two twisted $NbSe_2$ superconducting-diode devices with nominal twist angles of 1°. Each device contains four electrical contacts connected to the top $NbSe_2$ layer and another four contacts connected to the bottom layer, allowing the bottom layer, top layer, and overlap junction region to be measured independently.

Figure S4a shows the in-plane magnetic-field dependence of I_C^+ and $|I_C^-|$ for the bot-

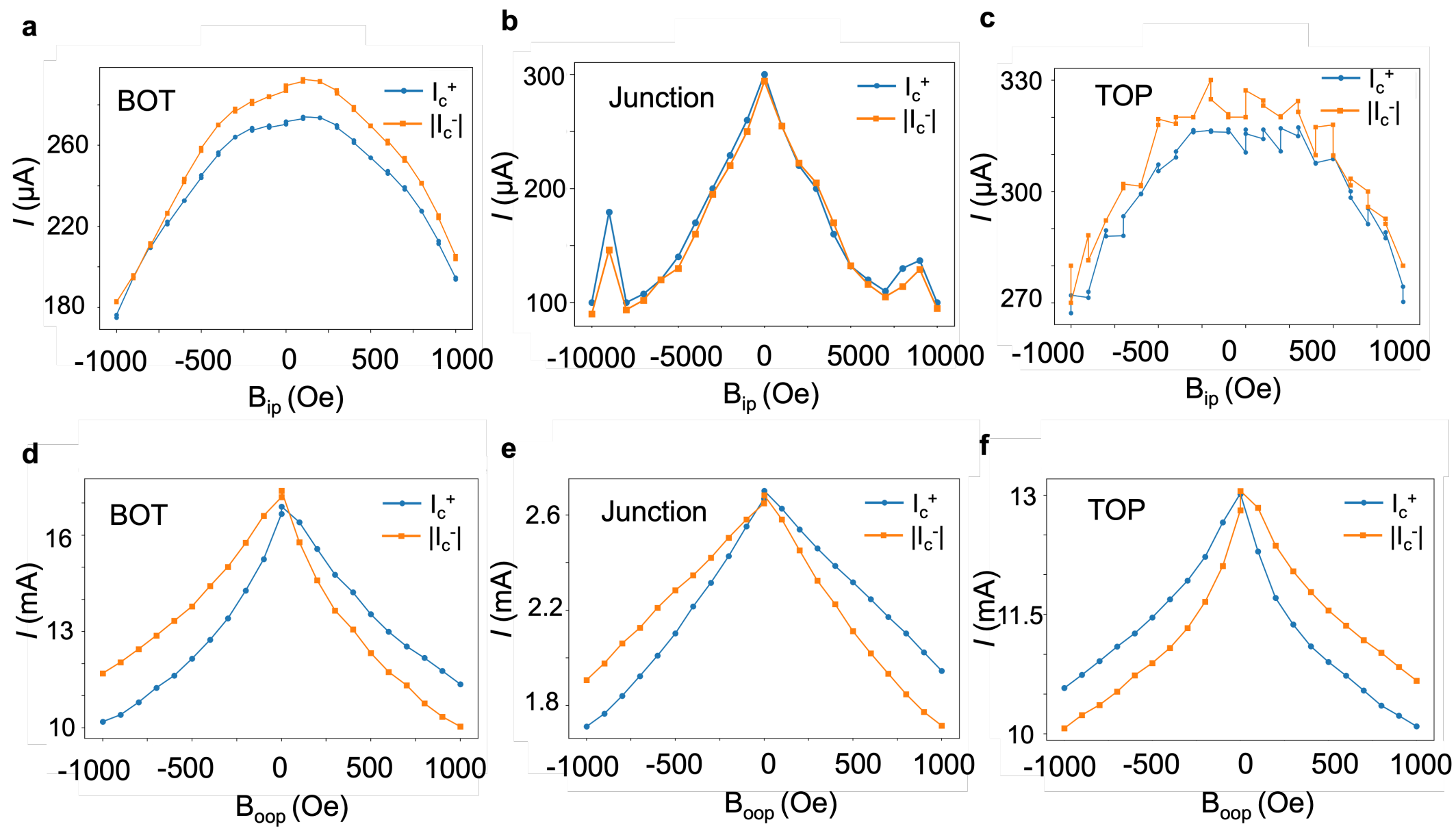

Figure S4: In-plane and out-of-plane magnetic-field dependence of the critical currents in the individual $NbSe_2$ layers and the overlap junction region for two twisted devices. a–c, Positive and negative critical currents, I_C^+ and $|I_C^-|$, measured in the bottom layer (**a**), overlap junction region (**b**), and top layer (**c**) of the first device as functions of the in-plane magnetic field, B_{ip}. The individual bottom and top layers exhibit broad field-dependent critical-current profiles, whereas the junction region shows a stronger and less monotonic variation over a substantially wider magnetic-field range. **d–f**, Corresponding measurements obtained from the bottom layer (**d**), overlap junction region (**e**), and top layer (**f**) of the second device. For this device, all three regions exhibit a maximum critical current near zero field and a gradual suppression with increasing $|\mathbf{B}_{ip}|$. The finite difference between I_C^+ and $|I_C^-|$ indicates nonreciprocal superconducting transport in both the individual layers and the overlap junction region.

tom layer of the first device. Both critical-current branches exhibit a broad maximum near zero field and decrease gradually as $|\mathbf{B}_{ip}|$ increases. Figure S4b presents the corresponding measurement for the overlap junction region. Compared with the individual layers, the junction exhibits a stronger and less monotonic dependence on $\mathbf{B}_{ip}$ over a substantially wider magnetic-field range. Figure S4c shows the response of the top layer, which has a larger overall critical current than the bottom layer while retaining a broad field-dependent profile.

Figure S4d shows the out-of-plane magnetic-field dependence of the critical currents for the bottom layer of the second device. The critical current reaches its maximum near zero field and is suppressed with increasing $|\mathbf{B}_{oop}|$. Similar behavior is observed in the overlap junction region and the top layer, as shown in Figs. S4e and S4f, respectively. In all measurement configurations, a finite separation between I_C^+ and $|I_C^-|$ is observed, demonstrating nonreciprocal superconducting transport in both the individual $NbSe_2$ lay-

ers and the overlap junction region.

Supplementary Note 4: QuTip simulation

The quantum simulation in this study employs the Quantum Toolbox in Python (QuTiP), a robust open-source software package tailored for simulating quantum systems [4]. QuTiP enables efficient numerical computation of quantum states, density matrices, Hamiltonians, and time evolution, making it well-suited for modeling complex systems such as the multi-qubit quantum diode examined here. In this work, QuTiP is used to represent and manipulate quantum states, implement time evolution operators, and incorporate realistic effects such as quantum noise and decoherence. The Josephson junction is modeled as the source of η within the system. Leveraging QuTiP's computational capabilities, the simulation systematically investigates forward and reverse quantum state transfer probabilities under varying levels of coupling asymmetry and noise, effectively capturing the dynamics critical to evaluating quantum diode performance.

Figure S5 presents QuTiP simulations that reveal the fundamental connection between transport dynamics and the underlying bound state structure in a twisted superconducting quantum diode. Figure S5a and S5b compare transport fidelity for values η. In the absence of noise (Fig. S5a), the system exhibits clean oscillatory patterns that reflect ideal unitary evolution. By contrast, the inclusion of realistic noise sources such as charge number fluctuations, superconducting phase decoherence, and Lindblad-type dissipation [5] (Fig. S5b) produces damping and irregularities at longer times, while the essential asymmetry between forward (solid) and reverse (dashed) transport remains intact across all efficiencies. To function as a qubit for quantum computing, a superconducting diode should ideally contain exactly two bound states in its potential well, a configuration that minimizes decoherence and enables precise state control.

As shown in Figs. S5c,e,g, achieving this two-state regime is dependent on both η, and the ratio of the Josephson to charging energy, $E_{\mathrm{J}}/E_{\mathrm{C}}$, where $E_{\mathrm{C}} = e^2/(2C)$ and $E_{\mathrm{J}} = \Phi_0 I_{\mathrm{C}}/2\pi$, with C the relevant circuit capacitance, $\Phi_0 = h/2e$ the flux quantum [3]. Our results for $E_{\mathrm{J}}/E_{\mathrm{C}} = 20$, Fig. S5c demonstrates that the two-state system is realized for efficiencies of $\eta \approx 22.9$–44.8% and 67.1–77.4%. The frequency analysis in Fig. S5d

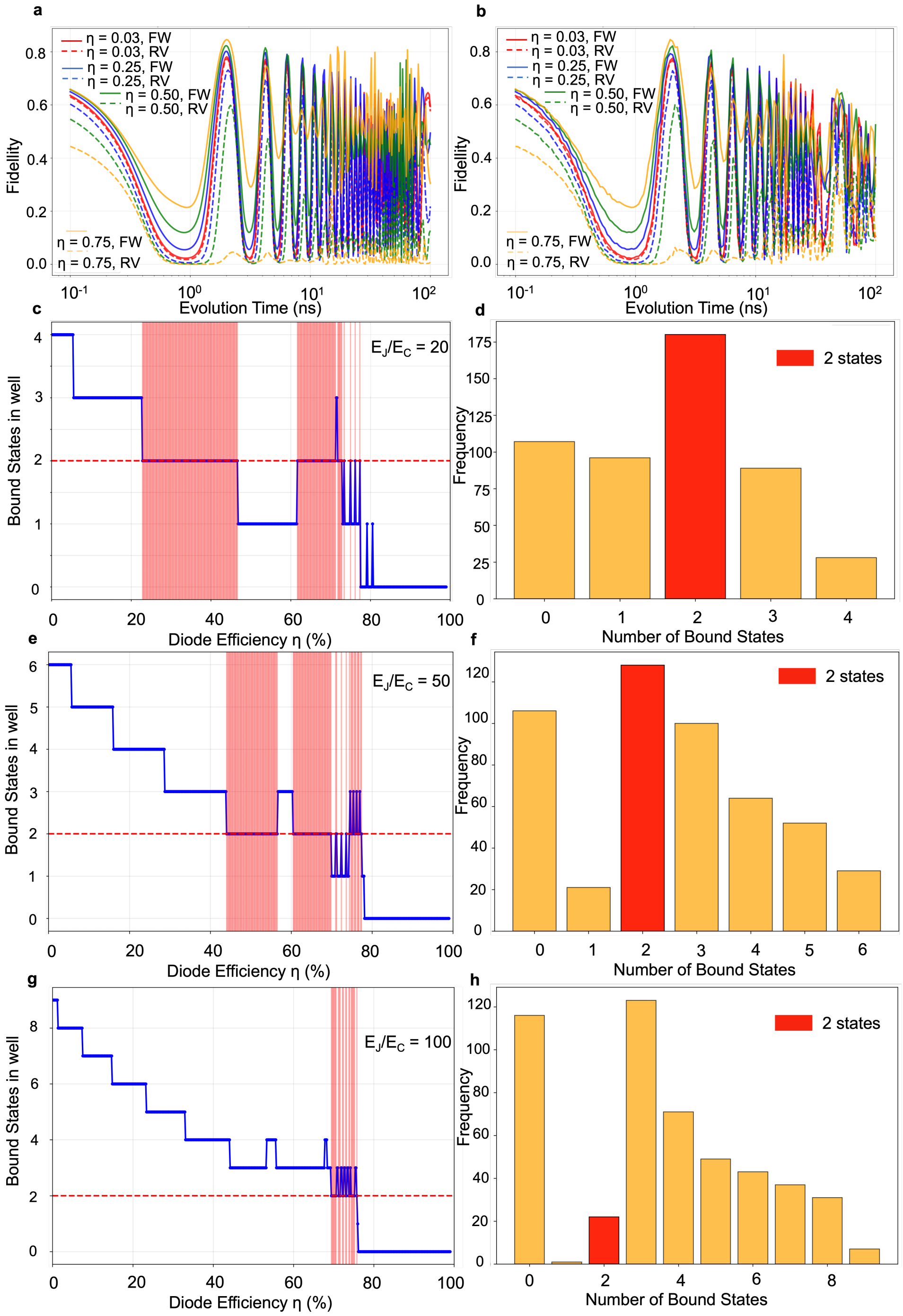

Figure S5: Quantum diode transport fidelity and bound-state analysis. (a) Forward (FW) and reverse (RV) transport fidelities as a function of evolution time for different diode efficiencies η in an ideal noise-free quantum system. (b) Forward and reverse transport fidelities as a function of evolution time for the same values of η as in (a), including charge noise, phase fluctuations, and dissipation. (c) Number of bound states in the potential well as a function of η for $E_{\mathrm{J}}/E_{\mathrm{C}} = 20$. (d) Frequency distribution of the number of bound states for $E_{\mathrm{J}}/E_{\mathrm{C}} = 20$. (e) Number of bound states in the potential well as a function of η for $E_{\mathrm{J}}/E_{\mathrm{C}} = 50$. (f) Frequency distribution of the number of bound states for $E_{\mathrm{J}}/E_{\mathrm{C}} = 50$. (g) Number of bound states in the potential well as a function of η for $E_{\mathrm{J}}/E_{\mathrm{C}} = 100$. (h) Frequency distribution of the number of bound states for $E_{\mathrm{J}}/E_{\mathrm{C}} = 100$. In (c), (e), and (g), the red dashed horizontal line marks the two-bound-state condition, and the red shaded regions indicate the ranges of η for which exactly two bound states are supported. In (d), (f), and (h), the red bars indicate configurations containing exactly two bound states.

confirms this two-state configuration is the most prevalent, with a count of 175. The analysis is extended for different $E_\mathrm{J}/E_\mathrm{C}$ ratios in Figs. S5e,g, which show how the η range for the two-state system shifts and narrows as the ratio increases. For $E_\mathrm{J}/E_\mathrm{C} = 50$, the range becomes $\eta \approx 44.0–77.4\%$, while for $E_\mathrm{J}/E_\mathrm{C} = 100$, it is $\eta \approx 69.4–75.8\%$. The corresponding histograms in Figs. S5f,h illustrate that the frequency of the two-state configuration changes to 120 and 20, respectively. Although its prevalence decreases with a higher $E_\mathrm{J}/E_\mathrm{C}$ ratio, the two-state regime, highlighted in red throughout the figures, remains a consistently identifiable and robust feature against parameter fluctuations. These results establish a clear and statistically significant operational window for engineering superconducting diodes as reliable building blocks for quantum information processing.

Supplementary References

1. Cao, Y., Luo, J., Fatemi, V., Fang, S., Sanchez-Yamagishi, J., Watanabe, K., Taniguchi, T., Kaxiras, E. & Jarillo-Herrero, P. Superlattice-Induced Insulating States and Valley-Protected Orbits in Twisted Bilayer Graphene. *Physical Review Letters* **117,** 116804 (2016).

2. Kim, Y., Herlinger, P., Moon, P., Koshino, M., Taniguchi, T., Watanabe, K. & Smet, J. H. Charge Inversion and Topological Phase Transition at a Twist Angle Induced van Hove Singularity of Bilayer Graphene. *Nano Letters* **16,** 5053–5059 (2016).

3. *Fundamentals and Frontiers of the Josephson Effect* (ed Tafuri, F.) (Springer Nature, Cham, 2019).

4. Johansson, J. R., Nation, P. D. & Nori, F. QuTiP: An Open-Source Python Framework for the Dynamics of Open Quantum Systems. *Computer Physics Communications* **183,** 1760–1772 (2012).

5. Breuer, H.-P. & Petruccione, F. *The Theory of Open Quantum Systems* (OUP Oxford, 2002).
