# Supplementary material for: Twisted superconducting quantum diodes for high fidelity anharmonic qubits
Source: Nat Commun. 2026 Oct 10;17:11503. doi: 10.1038/s41467-026-78376-1 (PMC13745581; doi:10.1038/s41467-026-78376-1)
Supplement: Supplementary file 2 [file 41467_2026_78376_MOESM2_ESM.pdf]

nature portfolio

Peer Review File

Twisted superconducting quantum diodes for high fidelity anharmonic qubit

Corresponding Author: Professor Yingying Wu

This file contains all reviewer reports in order by version, followed by all author rebuttals in order by version.

Version 0:

Reviewer comments:

Reviewer #1

(Remarks to the Author)
The manuscript by Han Zhong et al. reported the superconducting diode effect in a twisted NbSe2 device and explored its application toward superconducting qubit. In my opinion, the work represents a pioneering endeavor to apply twisted 2D materials for actual quantum circuits. As a prototype, while the device performance may be not perfect, the authors successfully demonstrated the working principles and the advantage of the van der waals heterostructure: the diode efficiency can be customized by controlling the twist angle. Given these merits, I would be happy to support the publication, but I do have several major and minor concerns listed below and I hope the authors can address them in a revised version.
1. In the abstract, it says “Conventional superconducting components lack directionality, causing energy loss and decoherence”. It is unfamiliar to me how the energy loss and decoherence are related to the lack of directionality. A counterexample: in the conventional BCS superconductor, with isotropic s-wave pairing (no directionality), electron pairs form a coherent state below Tc giving rise to zero dissipation.
2. The terms “twisted NbSe2 bilayers” and “twisted bilayer NbSe2” were used to describe the device with twisted layers consist of 16 to 60nm thick NbSe2 flakes. This is misleading because “twisted bilayer graphene” is generally used to describe two monolayer graphene flakes stacking together with a twist angle. I would suggest using simply “twisted NbSe2” instead as a more general yet accurate name.
3. Referring to Fig. 1c, Tc was marked as 6.87K, yet in this large range R-T curve, the number of data points obviously could not give reading with the accuracy of 0.01K. I am assuming this 6.87 number was obtained from another data to which it should be pointed.
4. “Fig. 1f, displays magnetochiral response” Fig. 1f displays the in-plane field response, from which we can see the robustness of the superconducting state under in-plain field, as a result of the strong Ising SOC. This is not related to magnetochiral response.
5. “Figure 2i summarizes η as a function of out-of-plane B for junctions of different thicknesses.” The word “junction” here is misleading since the device consists entirely of superconductor and does not consist a “junction”.
6. Speaking about junction, Fig 2h does show a typical hysteretic IV curve of a dc Josephson effect. Does this indicate a contaminated twisting interface of that 60nm device?
7. In Fig 2, if looked carefully, each curve is doubled. I am asuming they are associated with current swept back and forth. I may have missed it but I didn’t find a description. The authors should either describe the feature if they are related or remove the redundancy.
8. In Fig 2e, why are there so many jiggles in the orange curve?
9. Fig 3 shows that it needs a non-zero external field to have a non-zero superconducting diode effect. When applied to quantum computing circuit, does the external field affect the performance, for example, does it lead to decoherence?

(Remarks on code availability)

Reviewer #2

(Remarks to the Author)

The article presents experimental results on twisted $NbSe_2$ Josephson junctions showing superconducting diode (SD) effect with moderate efficiency below 30% and theoretical results showing that intermediate SD efficiency may be useful for interconnecting superconducting qubits. Overall, I find the paper interesting, particularly the theoretical part, where SDs are introduced into quantum circuits, but I believe that the experimental results and analysis are weak both in novelty and quality, so I cannot accept this paper for publication in its present form.

Since I am an experimentalist, I will focus my review on the experimental aspects of the work. My main points of criticism are:
1. The IV curves show a very smooth transition from superconducting to resistive state in combination with very small normal-state resistance. As a result, V offsets in the detector are clearly visible in the IV data, which makes me suspect about the possibility of having offsets in the current too.
a. Can the authors show a plot with abs(I) vs. abs(V) with corrected offsets showing that there is indeed an asymmetry which is not caused just by a current offset?
b. Can the authors show how the extracted SD efficiency depends on the voltage threshold used for the analysis to obtain the critical current, as shown here [P Moll, Communications Materials 6, 73 (2025)]?
2. The authors claim that there is an SD effect at zero magnetic field. How do they guarantee that the superconducting magnet of the PPMS has not trapped flux, resulting in an offset in the applied magnetic field? Typical offset values for PPMS setup with 9 T magnet are up to 20 Oe (see the Quantum Design Application Note 1070-207 "Using PPMS Superconducting Magnets at Low Fields"). This offset is consistent with Fig. 2l, which shows that the SD efficiency of the 16 nm sample undergoes a sign reversal at approximately 15 Oe under an out-of-plane magnetic field.
3. The in-plane magnetic field dependences on Fig.3 are also consistent with small out-of-plane magnetic field components causing the effect due to unintended misalignments of the sample with respect to the magnetic field. How are the samples aligned with the magnet? How accurate is the method?
5. The authors use the term 'quantum diode' referring to Ref. 14, however, the working principle of the diode proposed in Ref. 14 is quite different from the one they are describing in the text. Furthermore, they first use it in the abstract without a definition. I suggest that the authors consider using a name that is consistent with previous literature.
6. The authors use an asymmetric Bean-Livingston surface barrier as a means to explain their SD effect. Does this explanation imply that the critical current is dominated by the edges of the twisted $NbSe_2$ near the Josephson Junction (JJ)? What is the role of the JJ and the current-phase relation described extensively in the literature? Are there experiments showing what limits the critical current?
7. Why is the diode efficiency vs. magnetic field data fit to a sinusoidal function?
Minor points:
1. Error propagation is required when calculating the efficiencies from the critical currents.
2. The text immediately after Equation 1 is hard to follow because it mixes spin-valve device performance with superconducting diodes. "The push towards a high-diode efficiency is further motivated by the prospect that in van derWaals (vdW) heterostructures their atomically sharp interfaces could lead to high on/off ratios exceeding those in the common spintronic spin-valve devices."
3. Page 3 "We have fabricated twisted $NbSe_2$ structure using a twist angle of 1◦ since the reconstruction appears to be particularly strong when the twist angle is less than 1.2◦ [20]." What does this mean? How is this reconstruction related to the diode effect? This is the only occurrence of the word "reconstruction" in the paper.
4. Page 3 "Fig. 1f, displays magnetochiral response, further emphasising the role of external magnetic fields in tuning the electronic properties of the twisted $NbSe_2$ system." Magnetochiral anisotropy requires mapping the effect of an in-plane magnetic field in different directions in the plane, not just measuring in a single direction. Yet, these results likely indicate the anisotropy of superconductivity in layered $NbSe_2$. Does it break the Pauli limit? Is the anisotropy stronger than for non-twisted flakes? I also believe that the authors should refer to a reference relative to $NbSe_2$ Ising pairing, such as [X Xi et al. Nature Physics 12, 139 (2016).
5. Page 3: I cannot understand this sentence "Variations in the I-V curves indicate that inversion symmetry is broken in the superconducting state, while the observation of the diode effect along the current points, according to theory developed in [23], to the possibility of interfacial spin-orbit coupling texture with a non-zero radial Rashba component as also supported by density functional theory calculations in similar vdW materials with a twist [24, 25]."

(Remarks on code availability)

Reviewer #3

(Remarks to the Author)
Considering "Twisted superconducting quantum diodes: Towards anharmonicity and high fidelity", we found the key claims/results to be:

1. Theory/Simulations: Transmon qubit can be optimized by making use of nonreciprocal superconductors "superconducting diodes". The usual payoff for a transmon is flatness of the bands vs anharmonicity", ideally one could achieve both simultaneously, but the standard transmon increases one at the sacrifice of the other. In this work, the authors propose to sit in the flat band limit, $E_J/E_C>>1$, such that the transmon bands are flat, and thereby insensitive to charge noise. This comes at the price of removing the anharmonicity, and hence the qubit becomes susceptible to leakage out of the qubit subspace (since energy olevels become nearly equally spaced). But this is where the diode enters: it affords an additional handle in the transmon Hamiltonian that allows for anharmonicity. This appears to be the core idea, and the authors substantiate these claims using open quantum system simulations.

2. Experimental: On the experimental side, the authors design a new diode device via twisted NbSe2. Excitingly they find that twisted NbSe2 exhibits a small zero-field superconducting diode efficiency ~2%. Upon application of magnetic field, this can be raised to ~20%.

3. The suggestion is that: they find a decent candidate superconducting diode and they have simulations for such a diode placed into a transmon architecture. Together this strongly suggests a route to optimal qubits. We believe this is the logic of the paper.

We think the idea is novel and of interest to the readership of Nature Communications. Below we provide comments and concerns that should be addressed prior to potential publication.

Concerns:
1. The modelling is far too vague, e.g. Eq. (2) and surrounding text. What is the anomalous phase \phi_0, and how does it enter the modelling? What is f(\varphi), is it supposed to be F(\varphi)? In the equation for f(\varphi) the final term is just \eta, yet for F(\varphi) the final term is \eta\varphi.

2. What is the connection between diode efficiency \eta and the model parameter (also called \eta) that enters F(\varphi)? Is it one-to-one, and if so, can you please motivate this (I assume you could demonstrate this from I_c^+ and I_c^-).

3. You claim that by considering the functionality of a transmon qubit, the optimal diode efficiency is not 100%, but instead somewhere around 25%. Your device indeed has \eta~25%, but this is under applied field. Does your modelling/simulation take into account the existence of the applied field?

4. Can you clarify Fig. 4e – how to best understand the optimal range of \eta?

5. The experiments focus on a twist angle of approximately 1°. Is there a specific reason this particular angle is required to observe the diode effect? Do the authors expect similar nonreciprocal behavior at other twist angles, and under both in-plane and out-of-plane magnetic fields?

(Remarks on code availability)

Reviewer #4

(Remarks to the Author)
I co-reviewed this manuscript with one of the reviewers who provided the listed reports. This is part of the Nature Communications initiative to facilitate training in peer review and to provide appropriate recognition for Early Career Researchers who co-review manuscripts.

(Remarks on code availability)

Version 1:

Reviewer comments:

Reviewer #1

(Remarks to the Author)
The authors provided sufficient explanations to my questions, and made revisions to address my concerns. I am happy to support the publication in Nature Communications.

(Remarks on code availability)

Reviewer #2

(Remarks to the Author)
This work has two well-separated parts. The theoretical section shows an interesting analysis showing that a moderately asymmetric current phase relation can be optimal for making nonreciprocal qubits. The experiments show that twisted NbSe2 layers display a moderate diode effect up to 27.6%, which is consistent with the superconducting diode literature, including twisted van der Waals superconductors. However, several aspects of the experimental methodology remain insufficiently described, affecting both the reproducibility of the results and the ability to attribute the diode effect to the current-phase relation required to connect with theory. For these reasons, despite the novelty and interest of the theory part, I cannot recommend the paper for publication in Nature Communications in its present form.

1. Previous point 2: In the Methods section, it would be important to describe how the magnet was degaussed prior to the diode measurements. For instance, specifying the initial field, and oscillatory sweep ramp rate would allow readers to assess the magnetic-field environment and ensure reproducibility. Such information is necessary to rule out the possibility

that the observed field shift arises from trapped vortices in the superconducting magnet rather than from the diode effect itself.
2. Previous point 3: The authors state that the alignment between the magnet and the sample is accurate to 0.1°, but the manuscript does not describe how this alignment is achieved. Since the sample is glued to the holder, the tilt can already be larger than this value, and any rotator (including the PPMS high-resolution rotator) exhibits mechanical hysteresis that requires in-situ alignment in the magnetic field. For this reason, the procedure used to align the sample with respect to the magnetic field remains necessary. A description of the alignment protocol should be included in the Methods section to ensure reproducibility
3. It is well known from the twisted van der Waals literature that there is usually an uncertainty in the twist angle of the order of 0.1º. Since results from three different samples with different NbSe2 thicknesses (and possibly different twist angles due to experimental uncertainties) are reported and compared, a clear statement about twist angle uncertainty and how it affects the interpretation would be clarifying.
4. In the previous point 6, I asked whether the authors can provide measurements that clarify what limits the critical current in their device; the Josephson junction itself or vortex entry at the $NbSe_2$ edges. A straightforward way to establish this would be to measure the critical current of each individual flake and compare those values with the critical current measured across the junction. Such data would allow a more conclusive assessment of whether the observed diode effect originates from the current–phase relation of the Josephson junction (possibly combined with vortex physics, as the authors suggest) or whether it is instead dominated by vortex-related physics outside the junction. Could the authors include such measurements in the Supplementary Information?

(Remarks on code availability)

Reviewer #3

(Remarks to the Author)
The authors have provided detailed responses to many of the referees' comments. However, two of our previous questions remain insufficiently clarified.

Comment 1
In our previous report, we asked: "4. Can you clarify Fig. 4e – how to best understand the optimal range of η?"
Our wording may not have been sufficiently precise. The issue is the following: From Fig. 4e/f, the optimal fidelity appears to occur for η≈70–100%. However, the manuscript text suggests that the optimal regime is closer to η≈30%, which is particularly relevant since the reported devices operate near η≈30%.

The authors refer to Fig. 2d and appear to argue that for \eta\sim30%, exactly two levels are bound in the local well, and that this condition defines "optimal". It is not clear why the number of bound states alone should determine optimality. Leakage out of the two-level system is one of the key factors. And leakage can still occur into extended states. The primary diagnostic for leakage is the fidelity. This would imply optimal anisotropy of η≈70–100%.

We may be misunderstanding the authors' definition. To avoid ambiguity, could the authors clearly state how they define "optimal" in this context? If appropriate, it would also be helpful to indicate the optimal \eta range directly on Fig. 4e/f.

Comment 2
On the twist angle: the authors' response largely explains why small angles may be advantageous in generic moiré systems, but it does not clearly address whether \sim1^\circ is in any way special for the present system. While the general statement that small twist angles produce long-wavelength modulation and narrower moiré bandwidths is reasonable, the system under study here does not appear to be a conventional twisted bilayer composed of two independently rotated layers. It therefore remains unclear to what extent moiré reconstruction governs the bulk band structure in this case. The cited reference (Ref. [20]) discusses bilayer systems and does not directly establish that the same reasoning applies here. Likewise, the argument that enhanced interactions relative to the moiré bandwidth maximize the nonreciprocal response is supported by a reference to magic-angle twisted bilayer graphene, which is a rather different platform with distinct band topology and correlation physics.

If the choice of \sim1^\circ was primarily practical or heuristic (for example, based on general intuition from moiré physics or experimental convenience), it would be preferable to state this explicitly, either in the manuscript or at least in the response. Conversely, if the authors intend to claim that this angle is physically optimal for the observed diode effect, this should be supported by system-specific evidence or quantitative estimates, rather than by analogy to platforms with substantially different microscopic structure.

Comment 3
One additional question:
In Response Fig. R2, the diode efficiency in the plateau regime (for B=-90 Oe) appears to be approximately 10%. In the main text, a maximal efficiency of 26.7% is reported. Does this maximal value also correspond to a plateau regime, or does it occur under different conditions?

(Remarks on code availability)

Reviewer #4

(Remarks to the Author)
I co-reviewed this manuscript with one of the reviewers who provided the listed reports. This is part of the Nature Communications initiative to facilitate training in peer review and to provide appropriate recognition for Early Career Researchers who co-review manuscripts.

(Remarks on code availability)

Version 2:

Reviewer comments:

Reviewer #2

(Remarks to the Author)
In the revised version, the authors have improved the manuscript significantly.
However, I still have two important points left that need clarification:

1. Quantum Design recommends degaussing at 1-2 Tesla, not 100 mTesla (https://qdusa.com/siteDocs/appNotes/1070-207.pdf, page 9). Do the authors have independent evidence that the degaussing is complete? Otherwise, they will have to soften the claims about the zero-field diode performance.

4. The Bip scale is 10x higher for the Josephson junction than for the bottom and top layers, making the comparison very hard. Also, the density of points is very different. It would help the comparison if the junction data were in the same magnetic field range as the individual flakes. This would allow commenting whether Josephson Junction and bare flakes have a very different evolution with Bip or not in a more reliable way.

These two issues undermine the argument for a twist-angle-induced diode effect. The fact that the effect is also observed in the constituent layers suggests that the asymmetric Bean-Livingston surface barrier is a more plausible explanation.

(Remarks on code availability)

Reviewer #3

(Remarks to the Author)
We are now satisfied with the authors responses to our questions. The manuscript is, in our view, ready for publication.

(Remarks on code availability)

Reviewer #4

(Remarks to the Author)

(Remarks on code availability)

Version 3:

Reviewer comments:

Reviewer #2

(Remarks to the Author)
I am almost satisfied with the author’s responses and I thank them for the additional data.

I have personal experience trying to degauss such a magnet with small magnetic fields and being left with fields in the 10 Oe range. For this reason, I believe that it is not ruled out that the ~15 Oe offset in B or a significant part of it can be due to trapped flux from previous cooldowns, making the "nominal near-zero-field conditions" misleading. Unless a full outgassing from 1 T or above was done before starting the measurements, I believe that a note on a possible offset in B of several Oe should be added to the paper and shown in the figure or caption for clarity.

(Remarks on code availability)

Open Access This Peer Review File is licensed under a Creative Commons Attribution 4.0 International License, which permits use, sharing, adaptation, distribution and reproduction in any medium or format, as long as you give appropriate credit to the original author(s) and the source, provide a link to the Creative Commons license, and indicate if changes were made.
In cases where reviewers are anonymous, credit should be given to 'Anonymous Referee' and the source.
The images or other third party material in this Peer Review File are included in the article's Creative Commons license, unless indicated otherwise in a credit line to the material. If material is not included in the article's Creative Commons license and your intended use is not permitted by statutory regulation or exceeds the permitted use, you will need to obtain permission directly from the copyright holder.
To view a copy of this license, visit https://creativecommons.org/licenses/by/4.0/

Reply to Reviewers' Comments

We thank the Referees for their constructive and valuable comments, included below in their entirety *in blue*. We provide detailed responses to each comment *in black*, with the corresponding revisions to the manuscript highlighted in red. While all of our initial findings are retained, we are grateful for this opportunity to improve our presentation as well as the consistency of the used notations. For example, instead of B_{ip}, $B_{\text{in-plane}}$, and $B_{\|}$, we now only use B_{ip}. Similarly, F for fidelity is now changed to $\mathcal{F}$ to be distinct from F related to expressing the current-phase relation.

Reply to the Reviewer #1:

The manuscript by Han Zhong et al. reported the superconducting diode effect in a twisted $NbSe_2$ device and explored its application toward superconducting qubit. In my opinion, the work represents a pioneering endeavor to apply twisted 2D materials for actual quantum circuits. As a prototype, while the device performance may be not perfect, the authors successfully demonstrated the working principles and the advantage of the van der waals heterostructure: the diode efficiency can be customized by controlling the twist angle. Given these merits, I would be happy to support the publication, but I do have several major and minor concerns listed below and I hope the authors can address them in a revised version.

We are grateful for such a positive assessment of our work, which also reflects our intention to provide a presentation that may not show the best device performance, but could quickly stimulate further work by recognizing some overlooked aspects for superconducting diodes.

1. In the abstract, it says "Conventional superconducting components lack directionality, causing energy loss and decoherence". It is unfamiliar to me how the energy loss and decoherence are related to the lack of directionality. A counterexample: in the conventional BCS superconductor, with isotropic s-wave pairing (no directionality), electron pairs form a coherent state below T_C giving rise to zero dissipation.

We thank the Referee for the opportunity to clarify this potential confusion and modify our abstract. Indeed, the conventional s-wave BCS superconductor can be perfectly dissipationless without any "directionality" at low biases. Our statement instead was intended to address *circuit-level* properties, including losses and directionality. In superconducting quantum hardware, bidirectional microwave/supercurrent propagation leads to back-reflections and back-action (e.g., amplifier back-emission, impedance mismatches, parasitic resonances), which can re-inject noise into sensitive nodes, populate spurious channels, and thereby increase dissipation *elsewhere* in the circuit and cause qubit dephasing/relaxation. This is why practical cQED architectures typically require nonreciprocal components to protect qubits from the reflected noise. A superconducting diode provides an *intrinsic nonreciprocal element* that can reduce back-scattering pathways at the device level. We have rewritten the sentence in the abstract to avoid implying that a conventional BCS superconductor is dissipative:

"In superconducting circuits, bidirectional signal propagation can cause back-reflections and back-action that couple environmental noise back to coherent elements, degrading fidelity. Nonrecipro-

cal superconducting diodes provide an intrinsic route to suppress such back-scattering at the circuit level."

However, if this is not sufficient, we would welcome any suggestions for further improvements.

2. The terms "twisted $NbSe_2$ bilayers" and "twisted bilayer $NbSe_2$" were used to describe the device with twisted layers consist of 16 to 60nm thick $NbSe_2$ flakes. This is misleading because "twisted bilayer graphene" is generally used to describe two monolayer graphene flakes stacking together with a twist angle. I would suggest using simply "twisted $NbSe_2$" instead as a more general yet accurate name.

We thank the Referee for this important remark. While the term bilayer has been for decades used in superconducting junctions [for example, E. A. Demler et al., Phys. Rev. B **55**, 15174 (1997) or V. V. Ryazanov et al., Phys. Rev. Lett. **86**, 2427 (2001)] , we understand that, given the recent developments in van der Waals materials, the same term also has different meanings. In the revised manuscript we systematically replace "twisted bilayer $NbSe_2$"/"twisted $NbSe_2$ bilayers" by *"twisted $NbSe_2$"*.

3. Referring to Fig. 1c, T_C was marked as 6.87K, yet in this large range R-T curve, the number of data points obviously could not give reading with the accuracy of 0.01 K. I am assuming this 6.87 number was obtained from another data to which it should be pointed.

We appreciate the opportunity to clarify this issue since Fig. 1c does not convey the density of the taken data points. The reported value is extracted from the full, high-resolution $R - T$ dataset, rather than directly read from the coarse-scale plot in Fig. 1c. We consider a commonly used criterion [Pourret, A., Aubin, H., Lesueur, J., Marrache-Kikuchi, C., Berge, L., Dumoulin, L. & Behnia, K. Observation of the Nernst signal generated by fluctuating Cooper pairs. Nat. Phys. **2**, 683 (2006)] by which T_C is identified with the temperature at which the resistance is exactly at the half of the value found before the sharp drop begins (near 7 K). With 14 data points measured between 7 K and 6 K, where the resistance begins to drop toward zero, and the values very accurately recorded (with a small fraction of 0.01 K), we have then retained two decimal points to give $T_C = 6.87$ K, Accordingly, we have modified the description in Fig. 1 caption:
"(c) Dependence of the resistance R on temperature T indicating a critical temperature $T_C = 6.87$ K, which was extracted from high-resolution R–T data."

4. "Fig. 1f, displays magnetochiral response" Fig. 1f displays the in-plane field response, from which we can see the robustness of the superconducting state under in-plain field, as a result of the strong Ising SOC. This is not related to magnetochiral response.

We thank the Referee for noticing this issue. Indeed, the term "magnetochiral" was not correct. A strong Ising spin–orbit coupling locks electron spins out of plane, rendering the superconducting state insensitive to in-plane magnetic fields. We have accordingly modified the related text on p.3: "...Fig. 1f, displays anisotropic $\boldsymbol{B}_{\text{ip}}$ response, further emphasizing the role of external magnetic fields in tuning the electronic properties of the twisted $NbSe_2$ system. The enhanced resilience of superconductivity to $\boldsymbol{B}_{\text{ip}}$ fields in a few-layer $NbSe_2$, compared to $\boldsymbol{B}_{\text{oop}}$, is widely attributed to Ising spin-orbit coupling and Ising pairing [23].

Where we now added new Ref. [23] X. Xi et al., Nat. Phys. **12**, 139 (2016).

5. "Figure 2i summarizes η as a function of out-of-plane B for junctions of different thicknesses." The word "junction" here is misleading since the device consists entirely of superconductor and does not consist a "junction".

We appreciate this comment as indeed using the term "junction" can be unexpected. However, there are similar situations in previously published work where the presence of the vdW gap between two $NbSe_2$ layers acts as the barrier and the structure is described as a junction. This claim is supported by the published work in both $NbSe_2$/$NbSe_2$ and BSCCO/BSCCO vdW Josephson junctions Ref. [12]; Yabuki, N., Moriya, R., Arai, M., Sata, Y., Morikawa, S., Masubuchi, S. & Machida, T. Supercurrent in van der Waals Josephson junction. Nat. Commun. 7, 10616 (2016); Zhu, Y. et al. Presence of s-wave pairing in Josephson junctions made of twisted ultrathin $Bi_2Sr_2CaCu_2O_{8+x}$ flakes. Phys. Rev. X **11**, 031011 (2021). For example, artificially created vdW interface provides a sufficient decoupling of the wave functions of the two $NbSe_2$ crystals, with the vdW Josephson junction exhibiting a high supercurrent transparency [Yabuki, N., Moriya, R., Arai, M., Sata, Y., Morikawa, S., Masubuchi, S. & Machida, T. Supercurrent in van der Waals Josephson junction. Nat. Commun. **7**, 10616 (2016)]. In the BSCCO/BSCCO case, it was reported near the twist angle of 45°, that Josephson coupling was suppressed by the d-wave parameter of the material [12].

6. Speaking about junction, Fig 2h does show a typical hysteretic IV curve of a dc Josephson effect. Does this indicate a contaminated twisting interface of that 60 nm device?

Such a pronounced hysteresis observed in Fig. 2h for the 60 nm-thick device is a hallmark of an underdamped Josephson junction, rather than an indication of interface contamination. Due to the larger thickness, the junction exhibits a higher critical current I_C. Importantly, thinner devices fabricated using the identical twist and transfer procedure do not show pronounced hysteresis, indicating that the effect is geometrical.

We can expand this explanation using the standard resistively and capacitively shunted model of a junction: hysteresis is expected when the damping is weak, quantified by the Stewart-McCumber parameter $\beta_c = (2e/\hbar)I_C R^2 C > 1$. Increasing device thickness increases the effective capacitance C and, eventually, I_C, both of which enhance β_c and hence the hysteresis, even for clean interfaces. We add this explicit criterion and note that the retrapping current scales consistently with an underdamped junction regime. We also point out that the hysteresis is reproducible across the sweeps.

7. In Fig 2, if looked carefully, each curve is doubled. I am assuming they are associated with current swept back and forth. I may have missed it but I didn't find a description. The authors should either describe the feature if they are related or remove the redundancy.

Yes, this is an important observation. Each curve corresponds to forward and backward current sweeps to visualize hysteresis and switching. To clarify this we now add the bottom of p. 3:
"These curves, which correspond to the forward and backward current sweeps, nearly coincide in each of the panels."

8. In Fig 2e, why are there so many jiggles in the orange curve?

These small oscillatory features are attributed to intermittent phase-slips combined with local Joule heating. These oscillations do not affect the determination of the critical currents nor the extracted diode efficiency.

9. Fig 3 shows that it needs a non-zero external field to have a non-zero superconducting diode effect. When applied to quantum computing circuit, does the external field affect the performance, for example, does it lead to decoherence?

We thank the Referee for the opportunity to further address this point. We agree that a large global magnetic fields can be detrimental for many superconducting qubit platforms due to vortex entry and flux noise causing decoherence. Our key point is that *the circuit concept requires the nonreciprocal CPR (parameterized by η), but not a global applied field*. In the present transport experiments, B is simply a convenient tuning knob for η, while in our measured twisted $NbSe_2$ we observe $\eta \neq 0$, even when $B \to 0$.

“On p.5 we now add the following text:
For quantum-circuit integration there are multiple standard mitigation routes: (i) operating in a field-free diode regime (several vdW Josephson diodes exhibit $\eta \neq 0$ at $B \simeq 0$ [36]; (ii) using *local* on-chip bias lines/coils that generate the required flux only at the diode element while the qubit/resonator is shielded; (iii) using field-compatible qubit materials and vortex-pinning designs (e.g. flux-pinning holes) that allow coherent operation under mT-scale fields [37-39]; and (iv) using innovative qubit designs that are compatible with even larger fields ~ 0.1 T [40].”

For the purpose of this manuscript, it is also encouraging that there are independent efforts to advance the operation of transmon qubits, even at much higher applied filed than those at which we already observed maximum η. For example, this is discussed in Ref. [40], cited above J. Krause et al., Quasiparticle effects in magnetic-field-resilient three-dimensional transmons, Phys. Rev. Applied **22**, 044063 (2024), while it also shown that with finite B it is possible to elucidate decoherence contributions.

Reply to the Reviewer #2:

The article presents experimental results on twisted $NbSe_2$ Josephson junctions showing superconducting diode (SD) effect with moderate efficiency below 30% and theoretical results showing that intermediate SD efficiency may be useful for interconnecting superconducting qubits. Overall, I find the paper interesting, particularly the theoretical part, where SDs are introduced into quantum circuits, but I believe that the experimental results and analysis are weak both in novelty and quality, so I cannot accept this paper for publication in its present form.

We thank the Referee for finding our theoretical results interesting, which we expect could be also relevant to other systems supporting SD effect. We are grateful for the constructive comments and the opportunity to provide additional explanation of our experimental analysis.

Since I am an experimentalist, I will focus my review on the experimental aspects of the work. My main points of criticism are:

1. The IV curves show a very smooth transition from superconducting to resistive state in combination with very small normal-state resistance. As a result, V offsets in the detector are clearly visible in the IV data, which makes me suspect about the possibility of having offsets in the current too.
a. Can the authors show a plot with abs(I) vs. abs(V) with corrected offsets showing that there is indeed an asymmetry which is not caused just by a current offset?
b. Can the authors show how the extracted SD efficiency depends on the voltage threshold used for the analysis to obtain the critical current, as shown here [P Moll, Communications Materials 6, 73 (2025)]?

a. We thank the Referee for this important comment allowing us show a part of the analysis, supporting our findings, which was not previously included. We agree that offset effects must be carefully excluded given the smooth superconducting–resistive transition and the small normal-state resistance. To this end, the raw data are baseline-corrected according to

$$V_{\text{corr}} = V - V_0, \qquad I_{\text{corr}} = I - I_0, \tag{1}$$

where V_0 is determined from the near-zero-bias baseline and I_0 is constrained by the symmetry condition at $V_{\text{corr}} \approx 0$.

To directly test whether the observed nonreciprocity could arise from a spurious current offset, we plot the *folded* I–V characteristics, $|I_{\text{corr}}|$ as a function of $|V_{\text{corr}}|$, mapping $+I$ and $-I$ branches onto the first quadrant. This representation enables a direct comparison of the two transport branches under identical $|V_{\text{corr}}|$. As shown in FigR. 1, the two folded branches remain clearly separated after the offset correction, and the branch difference

$$\Delta|I| \equiv |I_+| - |I_-|, \tag{2}$$

remains finite and clearly visible over the analyzed bias range.

Importantly, a constant current offset would result only in a rigid shift of the I axis and cannot reproduce the observed voltage-dependent separation between the folded branches over an extended bias range. The persistence of the inequality

$$|I_C^+| \neq |I_C^-|, \tag{3}$$

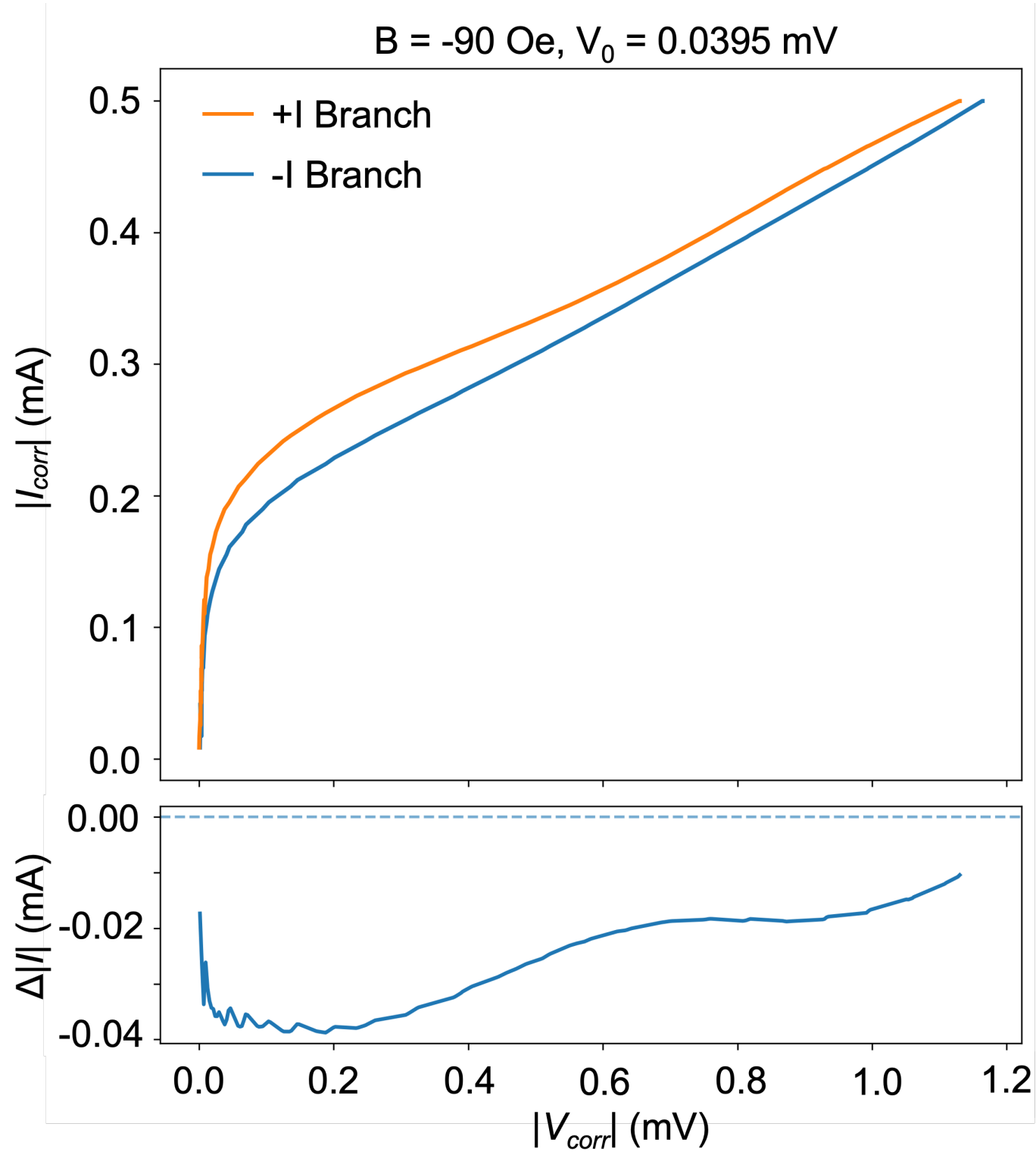

FigR. 1: Offset-corrected and folded I-V characteristics measured at $B = -90.08$ Oe. The upper panel shows the current $|I_{\rm corr}|$ as a function of the corrected voltage $|V_{\rm corr}|$ for the positive-current branch (blue) and negative-current branch (orange), after subtracting the residual voltage offset $V_0 = 0.03945$ mV and current offset $I_0 = 0$ mA. The folding procedure maps the $\pm I$ branches onto the first quadrant, enabling a direct comparison of transport asymmetry in the near-zero bias regime. The lower panel displays the difference $\Delta|I| = |I_+| - |I_-|$ as a function of $|V_{\rm corr}|$. Unlike the zero-field case, $\Delta|I|$ remains predominantly negative over the measured bias range, indicating a field-induced reversal of the transport asymmetry.

after offset correction,therefore, demonstrates that the observed nonreciprocity is intrinsic and not a detector artifact.

b. We thank the Referee for this helpful suggestions and have evaluated the robustness of the SD efficiency with respect to the threshold voltage $V_{\rm th}$ used to define the critical current, following the methodology of Moll *et al.* [24]. Which is included as the new Ref. [24]. For each chosen threshold, the polarity-dependent critical currents are defined by

$$|V_{\rm corr}(I_{\rm C}^{\pm})| = V_{\rm th}, \tag{4}$$

and the SD efficiency is computed as

$$\eta(V_{\rm th}) = \frac{|I_{\rm C}^{+}(V_{\rm th})| - |I_{\rm C}^{-}(V_{\rm th})|}{|I_{\rm C}^{+}(V_{\rm th})| + |I_{\rm C}^{-}(V_{\rm th})|}. \tag{5}$$

The resulting $\eta(V_{\rm th})$ is shown in FigR. 2, where it exhibits a stable, plateau-like behavior over a broad range of thresholds within the smooth transition regime. The values reported in the main text correspond to a threshold chosen within this plateau, and the residual variation of η across the plateau is included as a systematic contribution to the uncertainty.

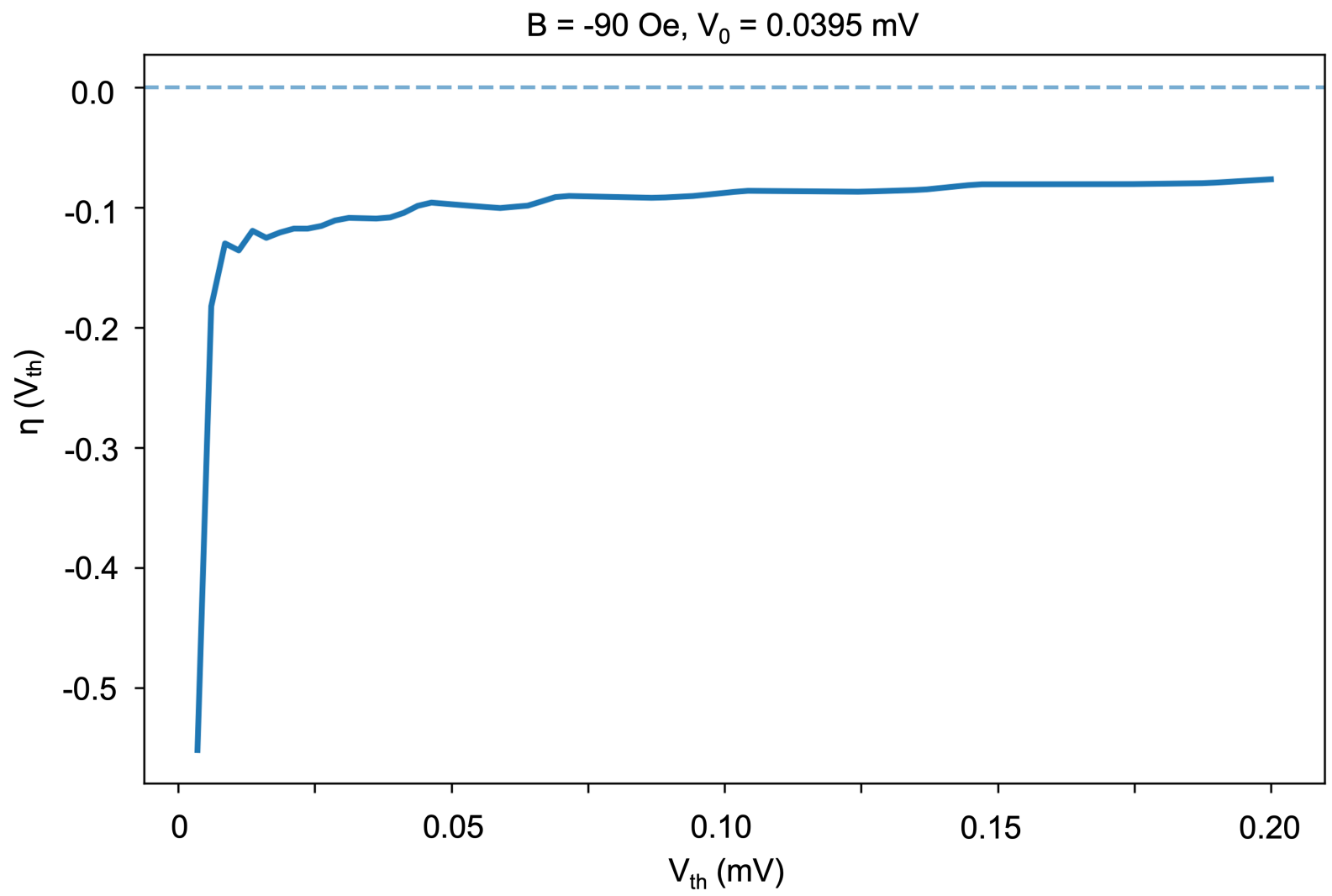

FigR. 2: Threshold voltage dependence of the SD efficiency $\eta(V_{\mathrm{th}})$ extracted at $B = -90.08$ Oe. For each threshold V_{th}, the polarity-dependent critical currents $I_{\mathrm{C}}^{\pm}$ are defined by $|V_{\mathrm{corr}}(I_{\mathrm{C}}^{\pm})| = V_{\mathrm{th}}$, following Ref. [24]. At very small thresholds, η varies strongly due to the smooth transition and finite voltage noise. Importantly, over a broad range of experimentally relevant thresholds, $\eta(V_{\mathrm{th}})$ exhibits a stable plateau, demonstrating that the reported diode efficiency is robust against the specific choice of voltage criterion.

2. The authors claim that there is an SD effect at zero magnetic field. How do they guarantee that the superconducting magnet of the PPMS has not trapped flux, resulting in an offset in the applied magnetic field? Typical offset values for PPMS setup with 9 T magnet are up to 20 Oe (see the Quantum Design Application Note 1070-207 "Using PPMS Superconducting Magnets at Low Fields"). This offset is consistent with Fig. 2I, which shows that the SD efficiency of the 16 nm sample undergoes a sign reversal at approximately 15 Oe under an out-of-plane magnetic field.

We thank the Referee for this comment since SD effect at zero magnetic field could be a surprise. We agree that trapped flux in superconducting magnets can lead to residual fields of order 10–20 Oe [Quantum Design, Using PPMS Superconducting Magnets at Low Fields. Application **1070**, 1 (2009)]. To mitigate this, all zero-field measurements were performed after magnet oscillation (degaussing) and field-cooling protocols recommended by Quantum Design. Furthermore, the zero-field diode efficiency remains finite even when sweeping symmetrically around the inferred offset field, indicating that the observed effect cannot be solely attributed to trapped flux. To further support that SD effect at zero magnetic field is not an artifact, we mention a possible origin for the orbital magnetism and broken time-reversal symmetry in twisted structures as well. Additionally, there are a number of similar reports for the intrinsic origin of such zero-field SD in other twisted structures [for example, Refs. [11,12]; H. D. Scammell et al., Theory of zero-field superconducting diode effect in twisted trilayer graphene, 2D Mater. **9**, 025027 (2022); J.-X. Hu et al., Josephson diode effect induced by valley polarization in twisted bilayer graphene, Phys. Rev. Lett. **130** 266003 (2023); J. H. Pixley and P. A. Volkov, Twisted nodal superconductors, arXiv:2503.23683].

3. The in-plane magnetic field dependences on Fig.3 are also consistent with small out-of-plane magnetic field components causing the effect due to unintended misalignments of the sample with

respect to the magnetic field. How are the samples aligned with the magnet? How accurate is the method?

We thank the Referee for this opportunity to address the potential role of misalignment on our measurements and give a quantitative estimate showing that misalignment cannot explain the magnitude of the in-plane response. With an angular error $\delta\theta \lesssim 0.1°$, an in-plane (ip) field $B_{\rm ip} = 200\,$Oe would produce an unintended out-of-plane (oop) component $B_{\rm oop} \simeq B_{\|} \sin\delta\theta \approx 0.35\,$Oe. In contrast, the oop field scale where the strongest vortex-diode response occurs is $\sim$ 10–15 Oe (Fig. 2f). To mimic $B_{\rm oop} \sim 13\,$Oe at $B_{\rm ip} = 200\,$Oe would require $\delta\theta \simeq \arcsin(13/200) \approx 3.7°$, far larger than our alignment tolerance. Therefore field misalignment alone cannot account for the observed in-plane-field dependence. We include this estimate in the revised manuscript on the bottom of p.4.

We note that the question # 4 was not originally included in the received report.

5. The authors use the term 'quantum diode' referring to Ref. 14, however, the working principle of the diode proposed in Ref. 14 is quite different from the one they are describing in the text. Furthermore, they first use it in the abstract without a definition. I suggest that the authors consider using a name that is consistent with previous literature.

We appreciate this opportunity to discuss the motivation for using the term quantum diode. We agree that the working principle in Ref. 14 is different from that in our work. In Ref. 14, nonreciprocal behavior is engineered through a time-modulated dimerized chain of microwave resonators coupled via superconducting flux qubits, where directionality emerges from adiabatic pumping of topological edge states. In our work, the term quantum diode is being use more in the context of "quantum bit diode". Specifically, we use *quantum diode* to mean a nonreciprocal Josephson element enabling *nonreciprocal coherent dynamics* (direction-dependent state transfer/fidelity) in a quantum superconducting circuit. We clearly distinguish our mechanism (intrinsic nonreciprocal Josephson element characterized by $|I_{\rm C}^+| \neq |I_{\rm C}^-|$) from the time-modulated topological pumping approach of Ref. 14. To avoid terminological ambiguity, we also adopt the phrase *"superconducting quantum-diode element"* or *"nonreciprocal Josephson element for quantum circuits"* throughout. While we wanted to retain previously used term quantum diode, just as there is a large number of very different mechanisms and implementations collectively named as the superconducting diode, we are open to further suggestions and the term changes from the Referees.

6. The authors use an asymmetric Bean-Livingston surface barrier as a means to explain their SD effect. Does this explanation imply that the critical current is dominated by the edges of the twisted $NbSe_2$ near the Josephson Junction (JJ)? What is the role of the JJ and the current-phase relation described extensively in the literature? Are there experiments showing what limits the critical current?

We thank the Referee for the opportunity clarify that there are *two* conceptually distinct steps: (i) The *equilibrium* nonreciprocal current-phase relation (CPR) sets the maximal dissipationless supercurrent; (ii) The *switching* from the superconducting to resistive state in transport measurements can additionally be influenced by vortex entry/exit barriers, heating, and edge current crowding. In

thin type-II superconducting weak links, the measured "critical current" can therefore be a *switching current* that depends on vortex dynamics as well as on CPR.

7. Why is the diode efficiency vs. magnetic field data fit to a sinusoidal function?

We appreciate this opportunity to clarify that the used sinusoidal form is an *empirical first-harmonic parametrization* to capture the observed odd-in-B trend of the SD efficiency η over the limited low-field window in Figs. 2-and-3. This is not a claim that the microscopic mechanism is *exactly* sinusoidal. Periodic dependence on magnetic flux is generic in Josephson interference and in field-tunable phase offsets; over a narrow field interval a single harmonic typically dominates, motivating $\eta(B) \approx a \sin(bB + c)$ as a compact representation.

In the manuscript, on p.5 we replaced "captures the fundamental physics" by "we use an empirical sinusoidal relationship with an applied B," and also note "this simple first-harmonic parameterization effectively captures the observed B-dependent diode behavior."

Minor points:

1. Error propagation is required when calculating the efficiencies from the critical currents.

We now explicitly provide the propagated uncertainty: for $\eta = (|I_{\mathrm{C}}^{+}| - |I_{\mathrm{C}}^{-}|)/(|I_{\mathrm{C}}^{+}| + |I_{\mathrm{C}}^{-}|)$ (with $|I_{\mathrm{C}}^{\pm}| > 0$),

$$\sigma_\eta = \frac{2}{(|I_{\mathrm{C}}^{+}| + |I_{\mathrm{C}}^{-}|)^2} \sqrt{(|I_{\mathrm{C}}^{-}|\, \sigma_{I^+})^2 + (|I_{\mathrm{C}}^{+}|\, \sigma_{I^-})^2},$$

where $\sigma_{I^\pm}$ are obtained from the I-V trace near the switching point. These calculated uncertainties are now marked in our revised Fig. 3.

Below we also include calculated uncertainties related to Figs. 1 and 2 (FigR. 3& 4). However, the previous size of the colors symbols are almost indistinguishable from the size of the error bars so these main text figures have not been revised, unless the Referee suggests that such a change would be better.

2. The text immediately after Equation 1 is hard to follow because it mixes spin-valve device performance with superconducting diodes. "The push towards a high-diode efficiency is further motivated by the prospect that in van derWaals (vdW) heterostructures their atomically sharp interfaces could lead to high on/off ratios exceeding those in the common spintronic spin-valve devices."

If we view spin-valves and superconducting diodes as common representatives of normal-state and superconducting spintronics, their respective common figures of merit, showing the difference between on/off states, have a similar functional form. However, we follow the Referee's suggestion and remove the spin-valve analogy. We have rewritten the related text to focus on superconducting-diode metrics only: "The form of Eq. (1) conveniently quantifies the asymmetry between I_{C}^{+} and I_{C}^{-} with many works aiming to maximize η and the corresponding rectification [1, 2]." In the next paragraph we also expand the text to clarify: "However, how the diode efficiency influences much broader functionalities remains poorly understood, but it is essential to unlock their full potential. In quantum circuits the relevant objective can instead be maximizing fidelity/anharmonicity, subject to noise and design constraints."

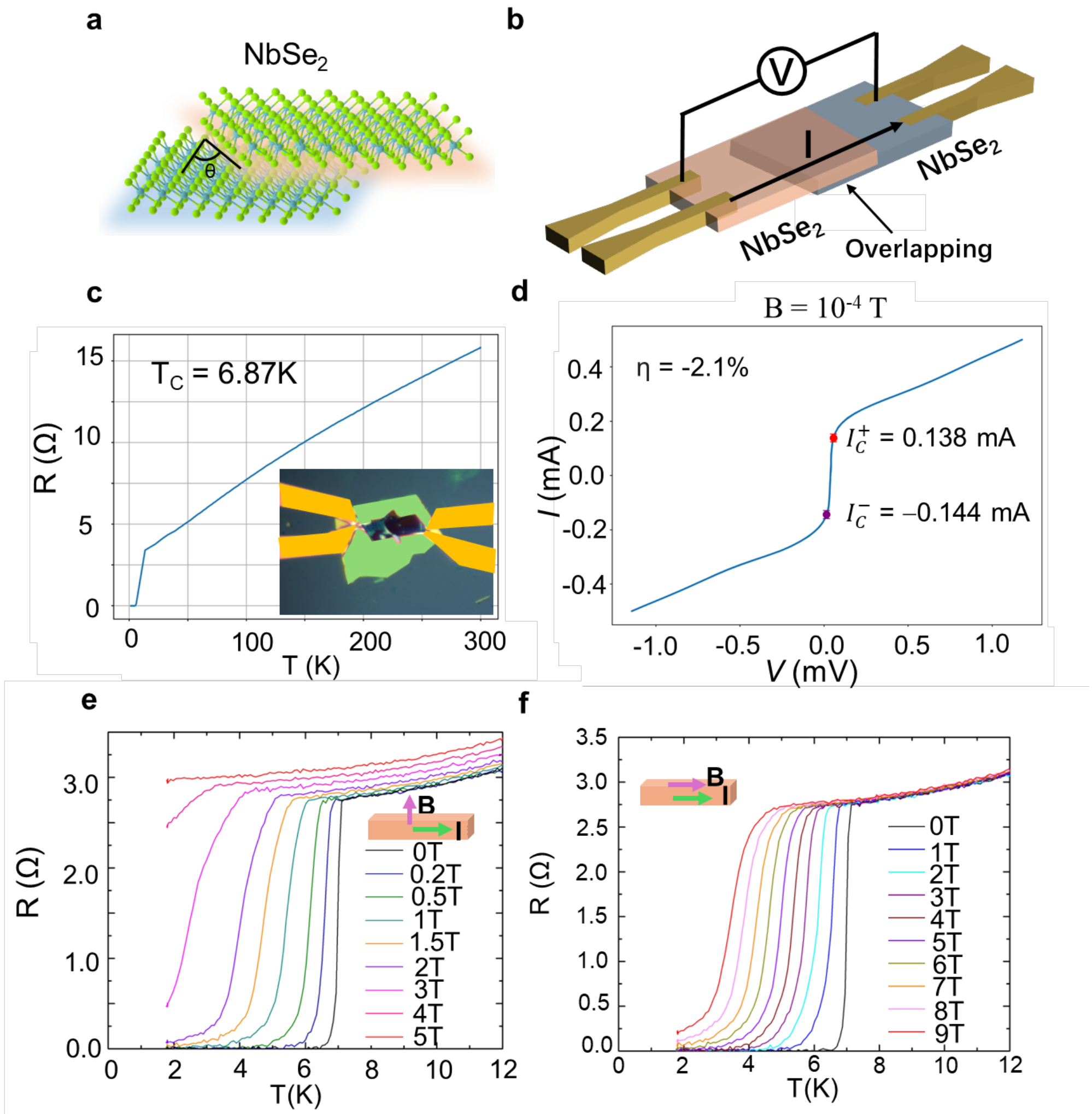

FigR. 3: Figure 1 from the main text. In (d) the added uncertainties accompany colored symbols for I_C^+, I_C^-.

3. Page 3 "We have fabricated twisted $NbSe_2$ structure using a twist angle of 1° since the reconstruction appears to be particularly strong when the twist angle is less than 1.2○ [20]." What does this mean? How is this reconstruction related to the diode effect? This is the only occurrence of the word "reconstruction" in the paper.

If it was not clear, by "reconstruction" we refer to the moiré-induced lattice and electronic reconstruction that can occur in small-angle twisted van der Waals heterostructures, where lattice relaxation leads to spatially varying stacking configurations and modified interfacial electronic structure. At very small twist angles, lattice relaxation produces extended commensurate stacking domains separated by solitonic boundaries; this *reconstruction* strongly modifies the interfacial electronic structure and can enhance inversion-symmetry breaking and interfacial spin-orbit textures, both of which promote a diode response. For marginally twisted $NbSe_2$, theory and experiments indicate especially strong reconstruction below $\sim 1.2°$ for certain registries [20]. This is why we focus on $\theta \approx 1°$.

In the main text on p.3 we added a few more sentence to better explain this situation and

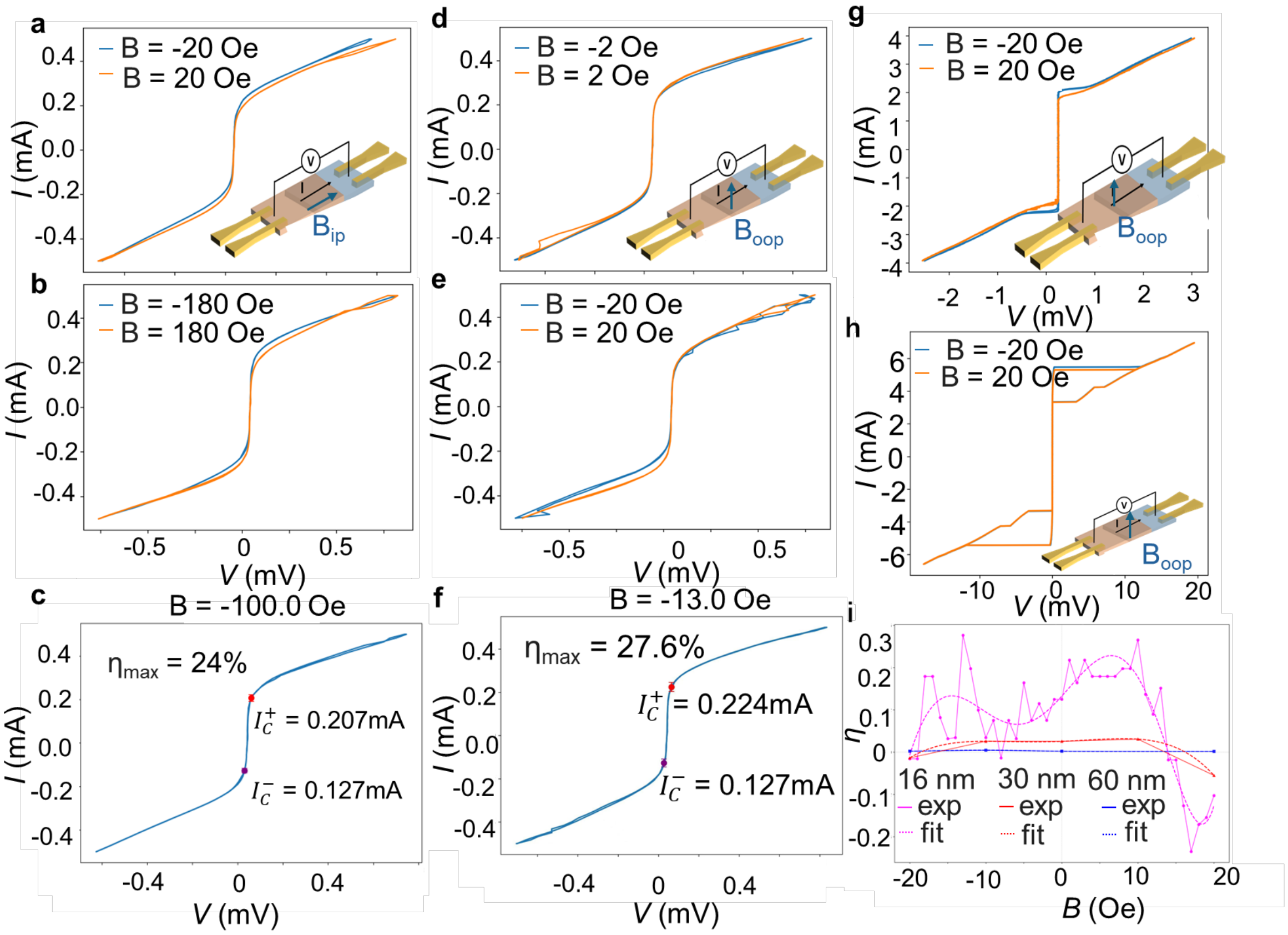

FigR. 4: Figure 2, main text. In (c,f) the added uncertainties accompany colored symbols for I_C^+, I_C^-.

included an additional reference [20].

[20] McHugh, J. G., Enaldiev, V. V. & Fal'ko, V. I. Moiré superstructures in marginally twisted $NbSe_2$ bilayers. Phys. Rev. B **108**, 224111 (2023).

We have also removed the previously used "reconstruction" by either (i) explaining it briefly at the first mention or (ii) replacing it by "moire-lattice relaxation and domain formation."

4. Page 3 "Fig. 1f, displays magnetochiral response, further emphasising the role of external magnetic fields in tuning the electronic properties of the twisted $NbSe_2$ system." Magnetochiral anisotropy requires mapping the effect of an in-plane magnetic field in different directions in the plane, not just measuring in a single direction. Yet, these results likely indicate the anisotropy of superconductivity in layered $NbSe_2$. Does it break the Pauli limit? Is the anisotropy stronger than for non-twisted flakes? I also believe that the authors should refer to a reference relative to $NbSe_2$ Ising pairing, such as [X Xi et al. Nature Physics 12, 139 (2016).

We agree that the wording "magnetochiral response" in the original manuscript was not correct. This is also addressed related to the comments from the Referee 1, point 4. A rigorous identification of magnetochiral anisotropy requires measuring the response for multiple in-plane field directions (and/or reversing the current relative to the field direction), whereas Figs. 1e-and-1f show the field dependence measured along a perpendicular and single in-plane direction. In the revised manuscript, we therefore remove the term "magnetochiral" and instead describe Fig. 1f as demonstrating an anisotropic in-plane-field response of superconductivity in layered $NbSe_2$.

Regarding the Pauli limit, our measurements in Fig. 1f are intended to highlight the qualitative field-tunability of the superconducting transport rather than to scrutinize the Pauli-limit violation. This violation is confirmed in other experiments on $NbSe_2$, including in an important work noted by the Referee, now cited as Ref. [23], X. Xi *et al.*, Nat. Phys. **12**, 139 (2016). Our extracted phase diagram for a bulk-like 60 nm-thick sample in FigR. 5, shows also the data from Ref. [23] and the potential to break the Pauli limit in thinner $NbSe_2$ samples.

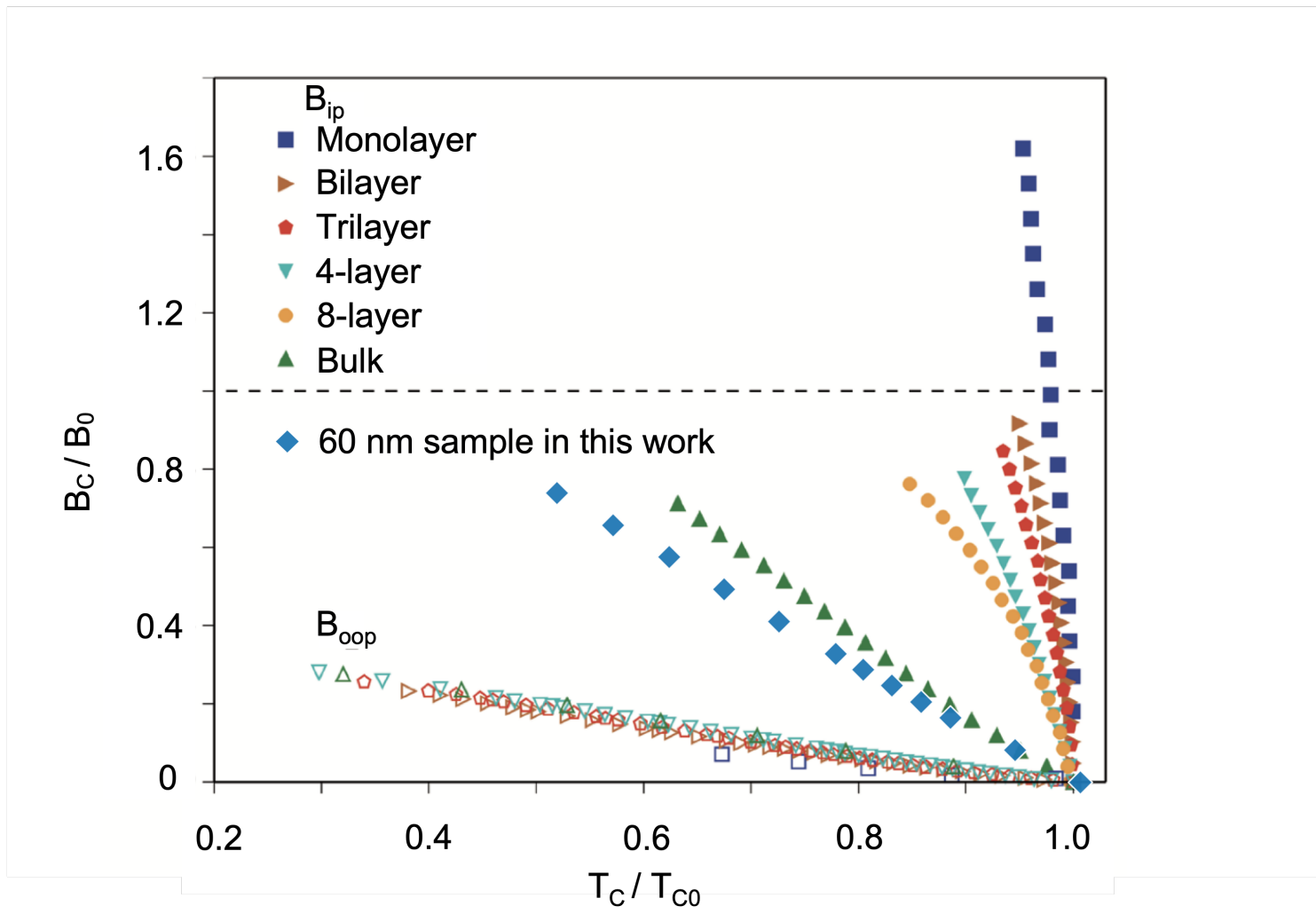

FigR. 5: Comparing upper critical magnetic fields H_{c2}/H_P from Ref. [23] with our measurements, normalized to the corresponding Pauli paramagnetic limit value, H_P, as a function of reduced critical temperature T_c/T_{c0} for samples with different thicknesses, ranging from monolayer to bulk. Solid symbols: in-plane magnetic fields, $H_{\|}$, open symbols: out-of-plane fields, $H_{\perp}$. The dashed horizontal line indicates H_P. The monolayer exhibits a pronounced enhancement of the in-plane $H_{c2} > H_P$, whereas increasing the layer number leads to a systematic reduction toward bulk-like behavior [23]. Blue diamonds denote the data in our work for the bulk-like 60 nm sample. For our device, $T_{c0} = 7.01$ K and $H_p = 12.89$ T, with a maximum $H_{c2}/H_P \approx 0.70$. Here we used the notation from Ref. [23].

While we do not have a sufficiently dense temperature-dependent data in thinner samples, comparing the bulk-like behavior of our 60 nm-thick and the counterpart from Ref. [23] provides a good agreement, especially if that other sample is even thinner that ours. In a future work, it would also be helpful to extend theoretical calculations to accurately include an emergent spin-orbit coupling form in the twisted $NbSe_2$ (such as the radial form of Rashba spin-orbit coupling) and how it modifies the thickness- and temperature-dependent evolution of H_{c2}.

The enhanced resilience of superconductivity to in-plane fields in few-layer $NbSe_2$ is widely attributed to Ising spin-orbit coupling and Ising pairing, corroborated also by the work in Ref. [23] suggested by the Referee. We expect an optimal twist angle for the anisotropy in the device. Similar nonreciprocal behavior may occur over a finite range of small twist angles where moiré effects remain significant, although the magnitude of the effect is expected to decrease as the twist angle increases and the moiré length scale shortens.

5. Page 3: I cannot understand this sentence “Variations in the I-V curves indicate that inversion symmetry is broken in the superconducting state, while the observation of the diode effect along the current points, according to theory developed in [23], to the possibility of interfacial

spin-orbit coupling texture with a non-zero radial Rashba component as also supported by density functional theory calculations in similar vdW materials with a twist [24, 25]."

We thank the opportunity to improve the readability of this sentence. The corresponding text on p. 4 is now rewritten to clearly separate: (i) The experimental observation of inversion-symmetry breaking inferred from the I-V characteristics and (ii) The possible microscopic origin of the superconducting diode effect discussed in the context of interfacial spin-orbit coupling.

We appreciate Referee's valuable guidance for these revisions and hope that we were able to address with them the main concerns.

Reply to the Reviewers #3 and #4:

Considering "Twisted superconducting quantum diodes: Towards anharmonicity and high fidelity", we found the key claims/results to be:

1. Theory/Simulations: Transmon qubit can be optimized by making use of nonreciprocal superconductors "superconducting diodes". The usual payoff for a transmon is flatness of the bands vs anharmonicity", ideally one could achieve both simultaneously, but the standard transmon increases one at the sacrifice of the other. In this work, the authors propose to sit in the flat band limit, $E_J/E_C \gg 1$, such that the transmon bands are flat, and thereby insensitive to charge noise. This comes at the price of removing the anharmonicity, and hence the qubit becomes susceptible to leakage out of the qubit subspace (since energy olevels become nearly equally spaced). But this is where the diode enters: it affords an additional handle in the transmon Hamiltonian that allows for anharmonicity. This appears to be the core idea, and the authors substantiate these claims using open quantum system simulations.

2. Experimental: On the experimental side, the authors design a new diode device via twisted NbSe2. Excitingly they find that twisted NbSe2 exhibits a small zero-field superconducting diode efficiency ∼2%. Upon application of magnetic field, this can be raised to ∼20%.

3. The suggestion is that: they find a decent candidate superconducting diode and they have simulations for such a diode placed into a transmon architecture. Together this strongly suggests a route to optimal qubits. We believe this is the logic of the paper.

We think the idea is novel and of interest to the readership of Nature Communications. Below we provide comments and concerns that should be addressed prior to potential publication.

We thank the Referees for their positive assessment of our work and succinct summary of findings. We appreciate the opportunity for improving our presentation with the provided suggestions.

Concerns: 1. The modelling is far too vague, e.g. Eq. (2) and surrounding text. What is the anomalous phase ϕ_0, and how does it enter the modelling? What is f(φ), is it supposed to be F(φ)? In the equation for f(φ) the final term is just η, yet for F(φ) the final term is $\eta\varphi$.

These are valuable questions and we are grateful for this opportunity to improve and expand the discussion related to our modeling and the background for Eq. (2). In addition to these comments, we also recognize some adjustments in our notation could improve clarity. For example, we now distinguish F from $\mathcal{F}$, used for fidelity. Since ϕ was used to describe one of states in expressing fidelity, the anomalous phase ϕ_0 is now replaced by φ_0, the same notation which is also used in A. Buzdin, Phys. Rev. Lett. **101**, 107005 (2008). We now explain ϕ_0, including a simple form how it appears, just as in the mentioned Phys. Rev. Lett. (2008).
"We model the diode element by an effective current-phase relation (CPR) $I(\varphi) = I_\mathrm{C} f(\varphi)$ and define the associated washboard potential $U(\varphi) = E_\mathrm{J} F(\varphi)$, such that $f(\varphi) = \partial_\varphi F(\varphi)$ and E_J is the Josephson energy [3]. The anomalous phase φ_0 describes the situation when $U(\varphi)$ attains the minimum at $\varphi = \varphi_0 \neq 0, \pi$, such that $I(\varphi_0) = 0$ [2], where a simple harmonic form of CPR is given by $f(\varphi) = \sin(\varphi + \varphi_0)$ [3]."

We hope that this text clarifies that $F(\varphi)$ describes the normalized phase-dependent (washboard) potential, while $f(\varphi)$ is defined as its derivative with respect to the superconducting phase

$$f(\varphi) = \partial F(\varphi)/\partial\varphi, \tag{6}$$

which corresponds physically to the normalized supercurrent. When expressed as a function of φ, any linear term in $F(\varphi)$ reduces to its coefficient upon differentiation. Consequently, a term $\propto \eta\varphi$ in $F(\varphi)$ gives rise to a constant contribution $\propto \eta$ in $f(\varphi)$. We also add this text on p. 6:
"While φ_0 is symmetry-allowed in the CPR, when inversion and time-reversal symmetries $\mathcal{P}$ and $\mathcal{T}$ are broken [2,3], it is irrelevant in the context of flux qubit. φ_0 just rigidly shifts $I(\varphi)$ and $U(\varphi)$, but has no direct impact on the diode nonreciprocity [2]. What matters is that $I(\varphi)$ becomes asymmetric due to a superposition of several (mutually shifted) harmonics, such that $\Delta I = |\max_\varphi I(\varphi)| - |\min_\varphi I(\varphi)| \neq 0$. Here we adopt a minimal parametrization, $f(\varphi) = \sin(\varphi - \arcsin\eta) + \eta$, that directly links η with ΔI following the underlying CPR $I(\varphi) = I_C f(\varphi)$; in general such a relation is highly nontrivial. The linear term in $F(\varphi)$ plays the same role as the standard "tilt" term in the washboard potential [3] and is used here as an effective description of the diode asymmetry at the circuit level (i.e. not as a statement about microscopic 2π-periodic Josephson energy)."

The modeling section, as also noted in our following answers, has been rewritten to better explain the physical meaning of each term and to establish a direct connection between the model parameters and the experimentally measured superconducting diode efficiency.

2. What is the connection between diode efficiency η and the model parameter (also called η) that enters F(φ)? Is it one-to-one, and if so, can you please motivate this (I assume you could demonstrate this from I_C^+ and I_C^-).

Indeed, the connection is one-to-one. However, that may have not been obvious, so we provide some related explanation.

$$f(\varphi) = \sin(\varphi - \delta) + \eta, \qquad \delta = \arcsin\eta,$$

the extrema are directly obtained because $\sin(\phi - \delta) \in [-1, 1]$:

$$I_\mathrm{C}^+ = I_\mathrm{C} \max_\varphi f(\varphi) = I_\mathrm{C}(1+\eta), \qquad I_\mathrm{C}^- = I_\mathrm{C} \min_\varphi f(\varphi) = I_\mathrm{C}(-1+\eta).$$

Hence $|I_\mathrm{C}^-| = I_\mathrm{C}(1-\eta)$ for $0 \leq \eta < 1$, and the diode efficiency becomes

$$\eta = \frac{|I_\mathrm{C}^+| - |I_\mathrm{C}^-|}{|I_\mathrm{C}^+| + |I_\mathrm{C}^-|} = \frac{(1+\eta) - (1-\eta)}{(1+\eta) + (1-\eta)} = \eta.$$

Therefore the model parameter in $F(\varphi)$ is exactly equal to the experimentally defined diode efficiency. We add this derivation to the Supplementary Information.

3. You claim that by considering the functionality of a transmon qubit, the optimal diode efficiency is not 100%, but instead somewhere around 25%. Your device indeed has η ~25%, but this is under applied field. Does your modelling/simulation take into account the existence of the applied field?

This is an important question and we thank the Referees for the opportunity to clarify our approach. While η generally varies with the applied filed B, in our simulations we treat η as the relevant parameter rather than modeling B explicitly. There are several motivations for this approach. Even though our experiments are focused on a specific superconducting diode twisted $NbSe_2$-based implementation with only a modest $\eta(B = 0) \sim 2\%$, there are other diode realizations where $\eta(B = 0) \neq 0$ and its magnitude could be even much larger. For example, Refs. [11,12]; H. D. Scammell et al., Theory of zero-field superconducting diode effect in twisted trilayer graphene; J.-X. Hu et al., Josephson diode effectinduced by valley polarization in twisted bilayer graphene, Phys. Rev. Lett. **130**, 266003 (2023); J. H. Pixley and P. A. Volkov, Twisted nodal superconductors, arXiv:2503.23683].

Furthermore, to observe the maximum η in our experiments only a modest $B \sim 10\,\mathrm{Oe}$ could be sufficient (Fig. 2f). At the same time, we have now added several new references [37-40] and the text on the bottom of p.5 to note various strategies on how qubits can retain their operation, even in the presence of a small or modest B.

We clarify in the manuscript that B is not an independent control parameter in the simulation because its only role is to tune the effective CPR (equivalently η). For quantum-circuit operation, the diode element can be realized either (i) in a field-free regime (several vdW Josephson diodes exhibit finite η at $B \simeq 0$ [36], or (ii) with a *local* on-chip bias field/flux line confined to the diode element, as is standard in superconducting circuits. We include this explicit "field-free or local-bias" implementation discussion to ensure the reader does not infer that global fields are required for the quantum diode concept.

4. Can you clarify Fig. 4e – how to best understand the optimal range of η?

We appreciate this question which has also motivated us to expand accordingly our description. While optimal range of η can depend on various parameters, for example, the ratio E_J/E_C, mentioned in the context of Fig. 4d, it is helpful to recognize that also for fidelity there is also an optimal η, instead of $\eta = 100\%$. In describing Fig. 4e we now add on p.7: "There is an optimal η as its increase has competing effects. A larger η: (i) enhances the CPR asymmetry, leading to improved directionality and stronger nonreciprocal transport, and (ii) tilts $U(\varphi)$ through the linear term in $F(\varphi)$, which reduces the effective potential well and suppresses desirable anharmonicity."

5. The experiments focus on a twist angle of approximately 1°. Is there a specific reason this particular angle is required to observe the diode effect? Do the authors expect similar nonreciprocal behavior at other twist angles, and under both in-plane and out-of-plane magnetic fields?

A special importance of small twist angle has already been recognized in Ref. 15 where in the case of grapehene it led to unconventional superconductivity. In our studies the twist angle of approximately 1° was chosen because it produces a long-wavelength moiré superlattice that strongly enhances electronic reconstruction [20]. and inversion-symmetry breaking, which are key ingredients for realizing a sizable superconducting diode effect. For $NbSe_2$-specific reconstruction results in Ref. 20 show particularly strong relaxation below $\sim 1.2^\circ$ for relevant registries. Near this small-angle regime, the moiré-induced band flattening and enhanced interactions maximize the nonreciprocal response. Discussed, for example, in Diez-Merida, J. et al. Symmetry-broken Josephson junctions and superconducting diodes in magic-angle twisted bilayer graphene. Nat.

Commun. **14**, 2396 (2023).

We do not expect the diode effect to be strictly limited to exactly 1°. Similar nonreciprocal behavior may occur over a finite range of small twist angles where moiré effects remain significant [as discussed in Carr, S., Fang, S. & Kaxiras, E. Electronic-structure methods for twisted moiré layers. Nat. Rev. Mater. **5**, 748 (2020). Diez-Merida, J. et al. Symmetry-broken Josephson junctions and superconducting diodes in magic-angle twisted bilayer graphene. Nat. Commun. **14**, 2396 (2023)], while the magnitude of the effect is expected to decrease as the twist angle increases and the moiré length scale shortens.

Reviewer #4 (Remarks to the Author):

I co-reviewed this manuscript with one of the reviewers who provided the listed reports. This is part of the Nature Communications initiative to facilitate training in peer review and to provide appropriate recognition for Early Career Researchers who co-review manuscripts.

We are grateful for these valuable comments from the Referees 3 and 4 which have helped us to provide a more complete description of our approach and modeling. We hope that these revisions have addressed the main concerns raised by the Referees.

Summary of Changes

The revisions are marked in red, including:

1. Modified abstract;
2. Improved notation for various quantities, including B_{ip}, B_{oop}, $\mathcal{F}$, φ_0;
3. Explained the significance of the small twist angle.
4. Expanded discussion of our modeling and simulation approach.
5. Included strategies to mitigate applied magnetic field, while retaining $\eta \neq 0$.
6. Added the calculated uncertainties in Fig. 3.
7. Added references [23,24,37-40] to corroborate our revisions.

Reply to Reviewers' Comments

We thank the Referees for their constructive and valuable comments, included below in their entirety *in blue*. We provide detailed responses to each comment *in black*, with the corresponding revisions to the manuscript highlighted in red.

Reply to the Reviewer #1:

Reviewer #1 (Remarks to the Author): The authors provided sufficient explanations to my questions, and made revisions to address my concerns. I am happy to support the publication in Nature Communications.

We are grateful for such a positive assessment of our work and for the Referee's guidance in improving our presentation.

Reply to the Reviewer #2:

Reviewer #2 (Remarks to the Author):

This work has two well-separated parts. The theoretical section shows an interesting analysis showing that a moderately asymmetric current phase relation can be optimal for making nonreciprocal qubits. The experiments show that twisted $NbSe_2$ layers display a moderate diode effect up to 27.6%, which is consistent with the superconducting diode literature, including twisted van der Waals superconductors. However, several aspects of the experimental methodology remain insufficiently described, affecting both the reproducibility of the results and the ability to attribute the diode effect to the current-phase relation required to connect with theory. For these reasons, despite the novelty and interest of the theory part, I cannot recommend the paper for publication in Nature Communications in its present form.

We thank the Referee for these comments which have also motivated our additional measurements. In this reply, all the questions were addressed. Specifically, we added the following revisions:

1. Proved the reproducibility of our measurements by carrying out fabrication and tests on two more twisted samples, with a diode efficiency reaching 20%-40%;
2. Carried out the measurements and proved the current-phase relation in these van der Waals junctions;
3. Added angle-dependent measurements to show how the alignment between electrical current and magnetic fields was made;
4. Clarified the angular resolution from the automated rotation stage in our transfer technique.

1. Previous point 2: In the Methods section, it would be important to describe how the magnet was degaussed prior to the diode measurements. For instance, specifying the initial field, and oscillatory sweep ramp rate would allow readers to assess the magnetic-field environment and ensure reproducibility. Such information is necessary to rule out the possibility that the observed

field shift arises from trapped vortices in the superconducting magnet rather than from the diode effect itself.

We appreciate the opportunity to clarify this point. Before the each measurement of the superconducting diode effects, the superconducting magnet was reset to minimize trapped magnetic flux. Specifically, the magnet was first ramped to a 100 mT and then brought back to zero field through several oscillatory sweeps with gradually decreasing amplitude. The field was ramped at a rate of approximately 10 mT/min during this procedure. Now this degauss procedure was added to Methods section.

2. Previous point 3: The authors state that the alignment between the magnet and the sample is accurate to 0.1°, but the manuscript does not describe how this alignment is achieved. Since the sample is glued to the holder, the tilt can already be larger than this value, and any rotator (including the PPMS high-resolution rotator) exhibits mechanical hysteresis that requires in-situ alignment in the magnetic field. For this reason, the procedure used to align the sample with respect to the magnetic field remains necessary. A description of the alignment protocol should be included in the Methods section to ensure reproducibility.

In our experiments, the sample was mounted on a rotatable probe compatible with the PPMS high-resolution rotator. We have carried out a 360° rotation of the sample both in plane and out of plane during the transport measurements at 10 K. We initially manually mount the drain and source direction along the magnetic field direction, $\mathbf{B}$. The minimum/maximum resistance indicating 0° offset in our mounting as shown in FigR. 1. These results confirm the accuracy of our alignment by showing a peak around 0° for rotating out of plane and a minimum for rotating in plane. This complements our prior estimate in the main text which shows the influence of the 0.1° error on the unintended out-of-plane B where, for clarity, we have replaced previous word "With" by "For."

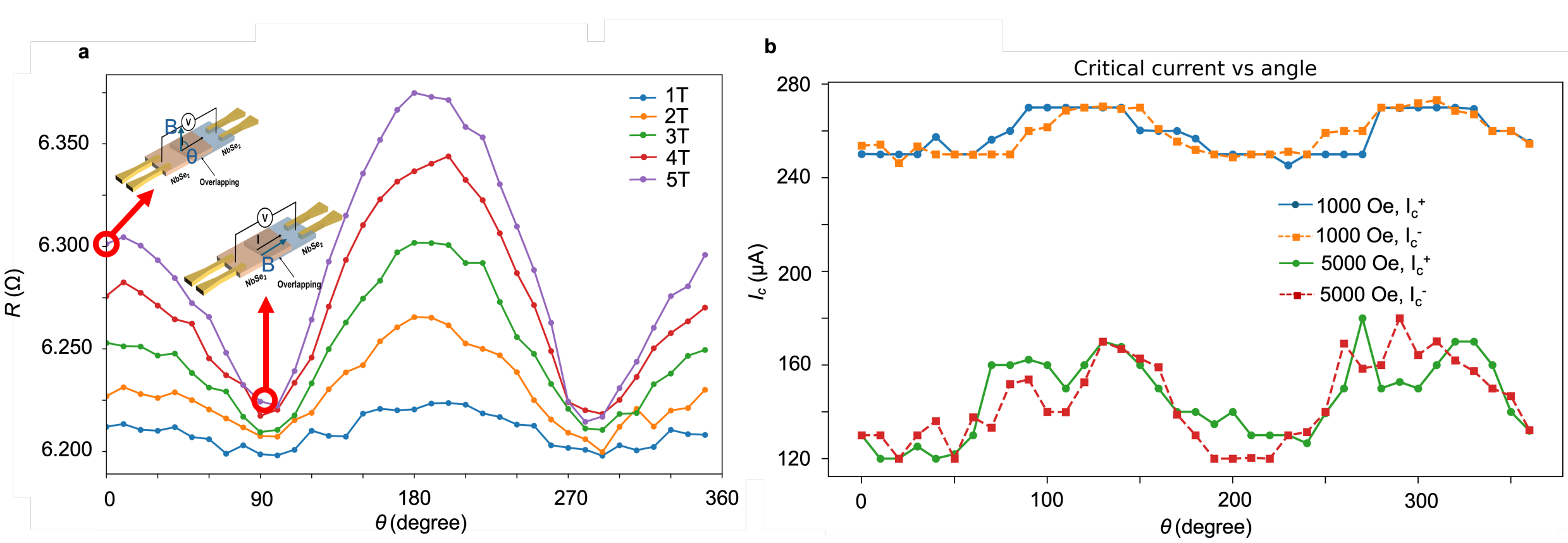

FigR. 1: **Angle dependence of the superconducting transport response under out-of-plane and in-plane magnetic fields. a**, Resistance R as a function of rotation angle θ measured at 10 K under different out-of-plane magnetic field. The curves show a clear angular modulation with characteristic extrema near $\theta \approx 0°$, 90°, 180°, and 270°, indicating dependence of the normal-state transport on field orientation. The inset schematics illustrate the device geometry and the definition of the rotation angle for the out-of-plane field configuration. **b**, Angle dependence of the positive and negative critical currents, I_C^+ and I_C^-, measured during in-plane field rotation at 2 K for representative B of 1000 Oe and 5000 Oe.

3. It is well known from the twisted van der Waals literature that there is usually an uncertainty in the twist angle of the order of 0.1°. Since results from three different samples with

different $NbSe_2$ thicknesses (and possibly different twist angles due to experimental uncertainties) are reported and compared, a clear statement about twist angle uncertainty and how it affects the interpretation would be clarifying.

We thank the Referee for this question. We used the "tear-and-transfer" method on the same flake. Our transfer stage was purchased from HQ Graphene and is equipped with a motorized rotation stage. This system provides a specified angular resolution of 0.003°, which is more than sufficient for accurately setting a twist angle of 1°. If the uncertainty is added, it would be 1°±0.003°, making negligible impact on the interpretation.

4. In the previous point 6, I asked whether the authors can provide measurements that clarify what limits the critical current in their device; the Josephson junction itself or vortex entry at the $NbSe_2$ edges. A straightforward way to establish this would be to measure the critical current of each individual flake and compare those values with the critical current measured across the junction. Such data would allow a more conclusive assessment of whether the observed diode effect originates from the current–phase relation of the Josephson junction (possibly combined with vortex physics, as the authors suggest) or whether it is instead dominated by vortex-related physics outside the junction. Could the authors include such measurements in the Supplementary Information?

We appreciate this additional explanation about the Referee's inquiry. To address this point we have fabricated a new superconducting diode with twist angle of 1° and performed additional measurements. Our device has four terminals connected to the top layer, as well as another four contacts to the bottom layer. This allows us to measure R-T and I-V characteristics. Figures 2a and 2b show I_C^+ and I_C^- as a function of in-plane B, B_{ip}, for each individual layers, i.e. top and bottom $NbSe_2$ layers. However, for the junction, I_C^+ and I_C^-, shown in FigR. 2c have a very different evolution with B_{ip}, from the individual layers.

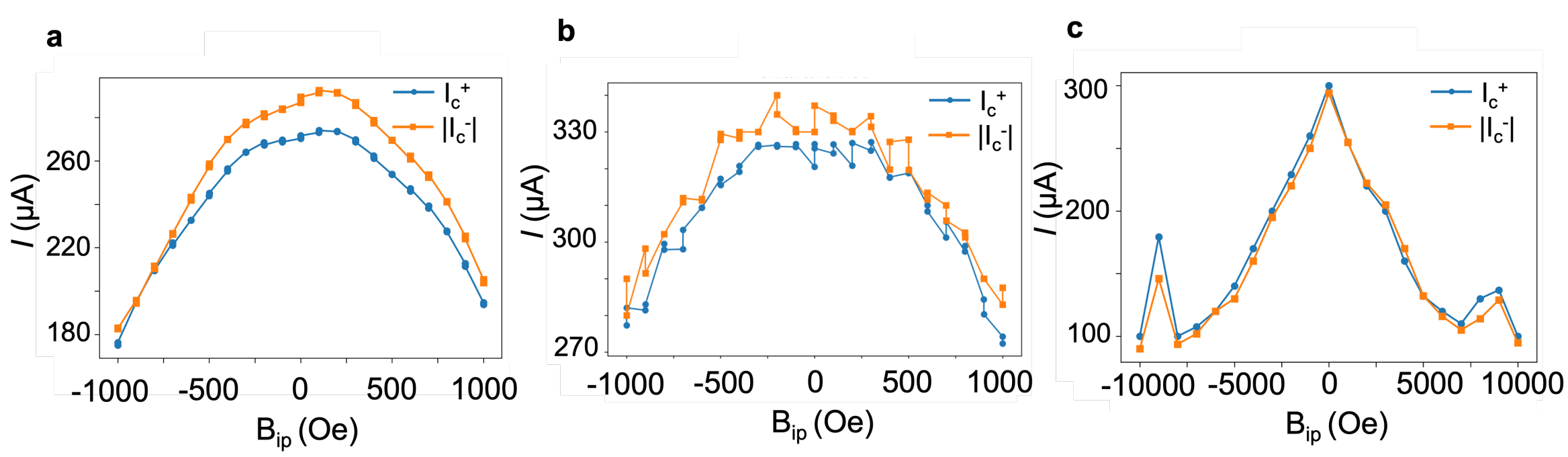

FigR. 2: **Magnetic-field dependence of the critical current for the individual $NbSe_2$ layers and the overlap junction region.** **a**, Positive and negative critical currents, I_C^+ and $|I_C^-|$, of the bottom-layer device as a function of the in-plane B_{ip}. The two branches show a parabolic dependence. **b**, Field dependence of I_C^+ and $|I_C^-|$ for the top-layer device. Compared with the bottom layer, the top layer exhibits a larger overall critical current while preserving a similar parabolic dependence on B_{ip}. **c**, Field dependence of I_C^+ and $|I_C^-|$ measured in the junction (overlap) region. In contrast to the single-layer devices, the junction displays a response centered near $B_{\text{ip}} = 0$, together with a more pronounced suppression of the critical current at larger B_{ip}, consistent with the junction-dominated superconducting transport.

To confirm that the underlying properties are compatible with Josephson junctions, as shown from the FigR. 3, which is now included in the main text as Fig. 1, we also map I-V characteristics

as B is varied, with the evolution of I_C described by

$$I_{\mathrm{C}}(B) = I_{\mathrm{bg}} + I_{\mathrm{C0}} \left| \frac{\sin[\pi(B - B_0)/B_p]}{\pi(B - B_0)/B_p} \right|, \tag{1}$$

where I_{bg} is a field-independent background contribution, I_{C0} is the amplitude of the Fraunhofer-like modulation, B_0 is the B offset corresponding to the center of the interference pattern, and B_p is the characteristic field period that determines the width of the central lobe and the spacing of the side lobes. The observed peak in I_{C} near $B = 0$ and its oscillatory suppression at larger B are consistent with Josephson-interference-like behavior [1] in the twisted $NbSe_2$ junction.

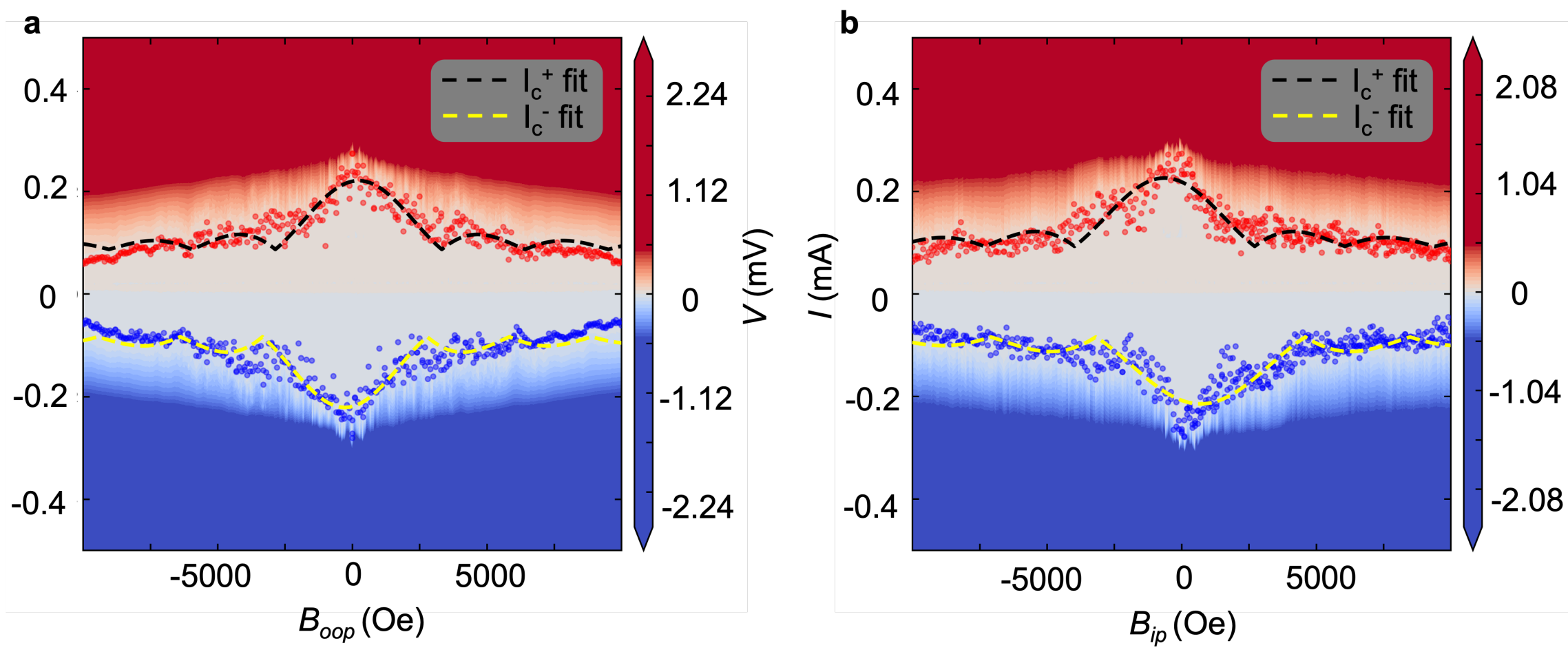

FigR. 3: Voltage maps as a function of I and B, (a) in plane and (b) out of plane, with the extracted I_{C}^+ (I_{C}^-), overlaid as red (blue) symbols. The black and yellow dashed lines represent the corresponding Fraunhofer-like fits to $I_{\mathrm{C}}^+(B)$ and $I_{\mathrm{C}}^-(B)$.

For panel (a), the fit to the positive branch yields $I_{C0}^+ = 1.333 \times 10^{-4}$ A, $I_{\mathrm{bg}}^+ = 9.257 \times 10^{-5}$ A, $B_0^+ = -628.95$ Oe, and $B_p^+ = 3346.74$ Oe, while the fit to the negative branch gives $I_{c0}^- = 1.305 \times 10^{-4}$ A, $I_{\mathrm{bg}}^- = 8.419 \times 10^{-5}$ A, $B_0^- = 707.35$ Oe, and $B_p^- = 3890.68$ Oe. For panel (b), the positive branch fit gives $I_{c0}^+ = 1.340 \times 10^{-4}$ A, $I_{\mathrm{bg}}^+ = 8.675 \times 10^{-5}$ A, $B_0^+ = 237.43$ Oe, and $B_p^+ = 3091.35$ Oe, whereas the negative branch yields $I_{c0}^- = 1.376 \times 10^{-4}$ A, $I_{\mathrm{bg}}^- = 8.363 \times 10^{-5}$ A, $B_0^- = -273.61$ Oe, and $B_p^- = 3057.96$ Oe. In both cases, the extracted critical currents exhibit a pronounced central lobe with side-lobe modulation, consistent with a Josephson-interference-like response. The finite offset field B_0 indicates that the interference maximum is shifted away from zero field, while the comparable fitting parameters for the positive and negative branches support the interpretation that the observed modulation originates from an intrinsic junction response in the device.

Reply to the Reviewers #3 and #4:

The authors have provided detailed responses to many of the referees' comments. However, two of our previous questions remain insufficiently clarified.

We thank the Referees for their suggestions on improving out mansuscript. In this revision, we made the following changes:

1. Clarified the emerging understanding of optimal diode efficiency in the context of qubits, fidelity, and anharmonicity;

2. Explained why 1° was picked as the twist angle and more future work needs to be carried out for a more comprehensive understanding;

3. Added more samples and measurements.

Comment 1

In our previous report, we asked: "4. Can you clarify Fig. 4e – how to best understand the optimal range of η?" Our wording may not have been sufficiently precise. The issue is the following: From Fig. 4e/f, the optimal fidelity appears to occur for η ≈70–100%. However, the manuscript text suggests that the optimal regime is closer to η ≈30%, which is particularly relevant since the reported devices operate near η ≈30%.

The authors refer to Fig. 2d and appear to argue that for η ∼30%, exactly two levels are bound in the local well, and that this condition defines "optimal". It is not clear why the number of bound states alone should determine optimality. Leakage out of the two-level system is one of the key factors. And leakage can still occur into extended states. The primary diagnostic for leakage is the fidelity. This would imply optimal anisotropy of η ≈70–100%.

We may be misunderstanding the authors' definition. To avoid ambiguity, could the authors clearly state how they define "optimal" in this context? If appropriate, it would also be helpful to indicate the optimal η range directly on Fig. 4e/f.

We thank the Referees for raising this important point which, with our work, will stimulate further studies. Indeed, in the context of superconducting diode effects, it is very challenging to define the term "optimal." However, this is in contrast to the prevailing understanding that $\eta \to 100\%$ should always be the most desirable. Following, the initial submission of our manuscript, we have asked precisely this question to some of the leading researchers in the field, including Teruo Ono, the author on an early work: "Observation of superconducting diode effect," Nature **584**, 373–376 (2020), and Stuart Parkin, the author of Ref. 35: "The field-free Josephson diode in a van der Waals heterostructure," Nature **604**, 653–656 (2022). Both of them have told us that $\eta \to 100\%$ should be universally desirable.

Unlike this prevailing understanding, we have tried to take a more nuanced approach where we mainly focus on the aspects related to the high fidelity, nonreciprocity, anharmonicity and qubit states. (1) Qubits: To enable qubits, it requires at least two bounds states. Please check Figure S3c in the Supplementary Information for $E_J/E_C = 20$, η within 0%-44.8% and 67.1%-77.4% can satisfy the requirement of two-level energy systems. This would exclude $\eta \to 100\%$ as the optimal value. (2) Fidelity: To enable unit fidelity, high efficiency is preferred while the intermediate efficiency can also allow high fidelity reading from Fig. 4e in the main text. This leaves out the efficiency of 0-∼25%. (3) Anharmonicity: from this consideration, we want a large energy interval difference between the neighboring levels. However, as shown in Fig. 4d in the main text, we have seen small ΔE different among 2-1 and 3-2 levels. We can further rule out the efficiency among 0-20% for having 3 or 4 bound states shown in Fig. S3c. Considering these three factors, our optimal η range for $E_J/E_C = 20$ is 25%-44.8% and 67.1%-77.4%.

Taken together, we are encouraged that our work will stimulate further studies which will advanced this seemingly simple question of what should be understood by the term "optimal."

Comment 2

On the twist angle: the authors' response largely explains why small angles may be advantageous in generic moiré systems, but it does not clearly address whether $\sim 1^\circ$ is in any way

special for the present system. While the general statement that small twist angles produce long-wavelength modulation and narrower moiré bandwidths is reasonable, the system under study here does not appear to be a conventional twisted bilayer composed of two independently rotated layers. It therefore remains unclear to what extent moiré reconstruction governs the bulk band structure in this case. The cited reference (Ref. [20]) discusses bilayer systems and does not directly establish that the same reasoning applies here. Likewise, the argument that enhanced interactions relative to the moiré bandwidth maximize the nonreciprocal response is supported by a reference to magic-angle twisted bilayer graphene, which is a rather different platform with distinct band topology and correlation physics.

If the choice of $\sim 1^\circ$ was primarily practical or heuristic (for example, based on general intuition from moiré physics or experimental convenience), it would be preferable to state this explicitly, either in the manuscript or at least in the response. Conversely, if the authors intend to claim that this angle is physically optimal for the observed diode effect, this should be supported by system-specific evidence or quantitative estimates, rather than by analogy to platforms with substantially different microscopic structure.

These are important questions and we are grateful for the opportunity to explain them further. In this work, the choice of a twist angle close to 1° should be understood primarily as a practical and heuristic choice, supported by literature showing exotic physics around this angle value. While we have not performed any theoretical calculations to select this angle, there is a supporting literature in various twisted systems showing that a small angle below 1.2 $^\circ$ producing long-wavelength structural modulation, which can modify the electronic environment and potentially influence non-reciprocal transport[2]. We note that other angles may also lead to similar effects, which is good considering this can contribute to universality of the effects we observed.

As the Referees correctly point out, the present system is not a conventional twisted bilayer composed of two independently rotated layers, and therefore the detailed role of moiré reconstruction in determining the bulk band structure remains an open question. Importantly, our conclusions do not rely on the assumption that $\sim 1^\circ$ is a special angle. Instead, we expect that similar behavior should occur for other small twist angles within the same regime. In other words, the key ingredient is operating in the twisted system, rather than a precise value of the twist angle.

In practice, an angle around 1° is experimentally convenient and relatively straightforward to realize reproducibly, which motivated our choice in this study. The essential point is that the diode effect emerges in the small-angle regime, and the exact value of 1° is not critical; exploring the systematic dependence on twist angle is an interesting direction for future work.

Comment 3

One additional question: In Response Fig. R2, the diode efficiency in the plateau regime (for B=-90 Oe) appears to be approximately 10%. In the main text, a maximal efficiency of 27.6% is reported. Does this maximal value also correspond to a plateau regime, or does it occur under different conditions?

We thank the Referees for this question which now can also be reexamined in the context of our newly fabricated samples with even higher diode efficiency noted in the Supplementary Information. In the main text, our reported maximal efficiency of 27.6% occurs at a different magnetic field (that what was provided in the response), where the asymmetry between the positive and negative critical currents is largest. In that case, the same analysis procedure was applied.

Therefore, the difference between the $\sim 10\%$ plateau in Fig. R2 and the maximal value reported in the main text simply reflects the field dependence of the diode efficiency rather than a difference in the extraction method.

Reviewer #4 (Remarks to the Author):

I co-reviewed this manuscript with one of the reviewers who provided the listed reports. This is part of the Nature Communications initiative to facilitate training in peer review and to provide appropriate recognition for Early Career Researchers who co-review manuscripts.

We are grateful for these valuable comments from the Referees which have helped us to provide a more complete description of our approach and modeling. We hope that these revisions have addressed their main concerns.

References

1. Yabuki, N., Moriya, R., Arai, M., Sata, Y., Morikawa, S., Masubuchi, S. & Machida, T. Supercurrent in van der Waals Josephson junction. *Nature Communications* **7,** 10616 (2016).
2. McHugh, J. G., Enaldiev, V. V. & Fal'ko, V. I. Moiré superstructures in marginally twisted $NbSe_2$ bilayers. *Physical Review B* **108,** 224111 (2023).

Reply to Reviewers' Comments

We thank the Referees for their constructive and valuable comments, included below in their entirety *in blue*. We provide detailed responses to each comment *in black*, with the corresponding revisions to the manuscript highlighted in red.

Reply to the Reviewer #2:

Reviewer #2 (Remarks to the Author): In the revised version, the authors have improved the manuscript significantly. However, I still have two important points left that need clarification:

We are grateful for such a positive assessment of our work and for the Referee's guidance in improving our presentation.

1. Quantum Design recommends degaussing at 1-2 Tesla, not 100 mTesla (https://qdusa.com/siteDocs/appNotes/1070-207.pdf, page 9). Do the authors have independent evidence that the degaussing is complete? Otherwise, they will have to soften the claims about the zero-field diode performance.

We thank the Referee for these comments which have also motivated our additional measurements. We agree that our earlier note could give the impression that the residual magnetic field was completely eliminated by the 100 mT degaussing procedure. Without an independent calibrated local-field measurement at the sample position, such a claim would be too strong. Following the Referee's suggestion, we have therefore revised the manuscript to avoid overclaiming a strictly zero-field diode effect. We now refer to this condition as "nominal near-zero-field conditions" rather than absolute zero field. This change was highlighted in red in the main text.

The reason we used a 100 mT degaussing field is that, in most cases, 100 mT was the maximum magnetic field applied during the experimental sequence. Therefore, this procedure was intended to remove the field history associated with the actual measurement range, without exposing the device to a substantially larger magnetic field than used in the experiment. We agree that this does not by itself prove that the residual field is completely removed. We have clarified this point in the revised manuscript and softened the corresponding claims.

4. The $B_{\rm oop}$ scale is $10\times$ higher for the Josephson junction than for the bottom and top layers, making the comparison very hard. Also, the density of points is very different. It would help the comparison if the junction data were in the same magnetic-field range as the individual flakes. This would allow commenting whether the Josephson junction and bare flakes have a very different evolution with $B_{\rm oop}$ or not in a more reliable way.

These two issues undermine the argument for a twist-angle-induced diode effect. The fact that the effect is also observed in the constituent layers suggests that the asymmetric Bean-Livingston surface barrier is a more plausible explanation.

We thank the Referee for this helpful suggestion. We agree that the previous comparison was not sufficiently direct because the Josephson junction and the individual $NbSe_2$ flakes were plotted over different out-of-plane magnetic-field ranges and with different point densities. This is due to the overheating of the device during the measurement, making the more detailed data unattainable. In the revised manuscript, we have fabricated a new twisted device and replotted the critical currents measured in the bottom flake, overlapping Josephson junction region, and top flake

over the same B_{oop} range, from approximately -1000 to $+1000$ Oe, with comparable magnetic-field intervals, 100 Oe, as shown in Fig. R1. It is clear the overall effect is not a simple addition of the critical currents from two flakes.

Figure R1a shows the positive and negative critical-current magnitudes, I_{C}^{+} and $|I_{\text{C}}^{-}|$, measured in the bottom $NbSe_2$ layer. Both branches reach their maximum values near zero magnetic field and decrease as $|B_{\text{oop}}|$ increases. The relative ordering of I_{C}^{+} and $|I_{\text{C}}^{-}|$ reverses across zero field, demonstrating a finite field-dependent nonreciprocal response in the bottom flake.

Figure R1b presents the corresponding results for the overlapping Josephson junction region. The junction critical current also reaches a maximum near zero field and decreases with increasing $|B_{\text{oop}}|$. However, the junction shows a different absolute critical-current scale and a different evolution of the separation between I_{C}^{+} and $|I_{\text{C}}^{-}|$ compared to the bottom and top flakes.

Figure R1c shows the results measured in the top $NbSe_2$ layer. Similar to the bottom layer, both critical-current branches decrease with increasing $|B_{\text{oop}}|$, while their relative ordering reverses across zero field. Nevertheless, the magnitude and field dependence of the branch separation differ from those observed in both the bottom layer and the overlapping junction region.

The revised comparison shows that a finite nonreciprocal response is present in both individual $NbSe_2$ flakes. The overlapping junction region, however, exhibits a distinct critical-current scale and magnetic-field evolution. These results suggest the twisted Josephson interface further modifies the magnitude and field dependence of the superconducting diode response. This data has been added to Supplementary Note 3 and Figure S4 in the Supplementary information.

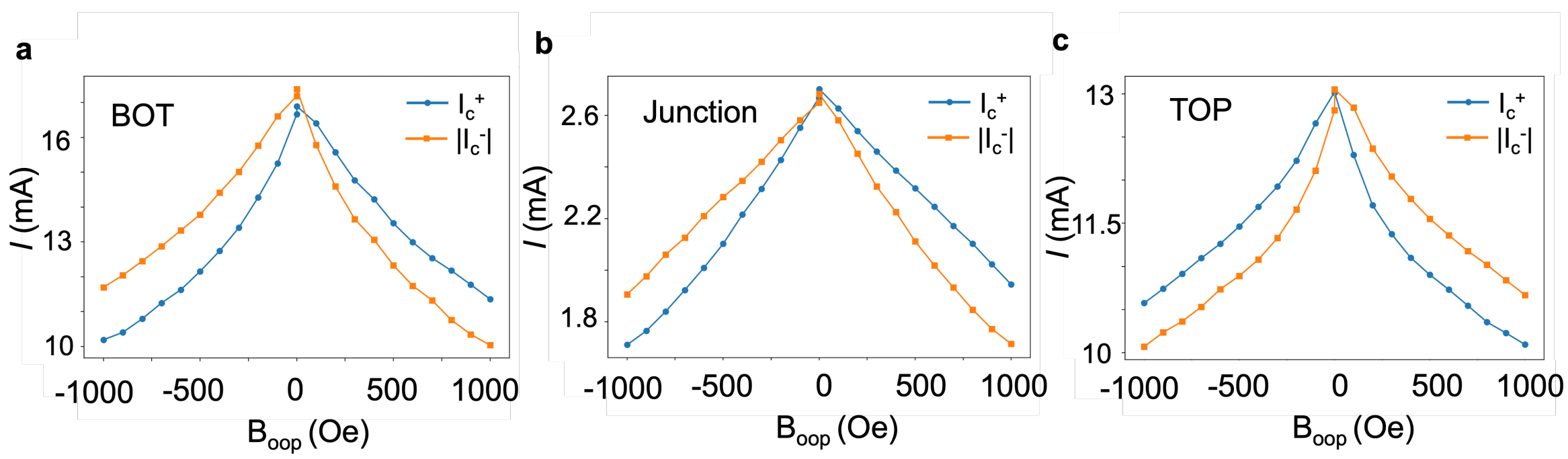

FigR. 1: **Out-of-plane magnetic-field dependence of the critical current in the individual $NbSe_2$ layers and the overlapping Josephson junction region.** **a**, Positive and negative critical-current magnitudes, I_{C}^{+} and $|I_{\text{C}}^{-}|$, measured in the bottom $NbSe_2$ layer as functions of the out-of-plane magnetic field B_{oop}. Both branches reach maximum values near zero field and decrease with increasing $|B_{\text{oop}}|$. Their relative ordering reverses across zero field, indicating a field-dependent nonreciprocal response. **b**, Corresponding I_{C}^{+} and $|I_{\text{C}}^{-}|$ measured in the overlapping Josephson junction region over the same magnetic-field range. The junction exhibits a maximum critical current near zero field and a suppression of both branches with increasing $|B_{\text{oop}}|$. Its critical-current scale and the field evolution of the two branches differ from those of the individual flakes. **c**, I_{C}^{+} and $|I_{\text{C}}^{-}|$ measured in the top $NbSe_2$ layer over the same B_{oop} range and with a comparable field-point density. The top layer also exhibits a reversal in the relative ordering of the two critical-current branches across zero field, although its critical-current magnitude and branch separation differ from those of the bottom layer and the overlapping junction. The direct comparison demonstrates that nonreciprocity is present in both individual flakes, while the overlapping Josephson junction exhibits a distinct magnitude and magnetic-field evolution of the superconducting transport.

Reply to the Reviewers #3:

We are now satisfied with the authors responses to our questions. The manuscript is, in our view, ready for publication.

We thank the Referees for a such a positive assessment and their valuable guidance which has helped us in improving our presentation.

Reply to Reviewer's Comments

We thank the Referees for their constructive and valuable comments, included below in their entirety *in blue*. We provide detailed responses to each comment *in black*, with the corresponding revisions to the manuscript highlighted in red.

Reply to the Reviewer #2:

Reviewer #2 (Remarks to the Author):

I am almost satisfied with the author's responses and I thank them for the additional data.

I have personal experience trying to degauss such a magnet with small magnetic fields and being left with fields in the 10 Oe range. For this reason, I believe that it is not ruled out that the 15 Oe offset in B or a significant part of it can be due to trapped flux from previous cooldowns, making the "nominal near-zero-field conditions" misleading. Unless a full outgassing from 1 T or above was done before starting the measurements, I believe that a note on a possible offset in B of several Oe should be added to the paper and shown in the figure or caption for clarity.

We thank the Reviewer for this helpful comment and for sharing their experience regarding the residual field of the superconducting magnet. We agree that, without a full degaussing procedure from a sufficiently high magnetic field prior to the measurements, a residual field of several Oe due to trapped flux cannot be completely excluded. Therefore, the observed ~ 15 Oe field offset may contain a contribution from the remanent field of the superconducting magnet, in addition to any sample-related contribution.

To avoid overinterpreting the absolute magnetic-field value, we have revised the manuscript by adding "We note that a residual magnetic field of several Oe due to trapped flux in the superconducting magnet cannot be completely excluded and may contribute to the observed field offset" following the near-Zero-field conditions in page 2 and in Figure 2 caption as well.
